# Neurofilaments in Sporadic and Familial Amyotrophic Lateral Sclerosis: A Systematic Review and Meta-Analysis

**DOI:** 10.3390/genes15040496

**Published:** 2024-04-16

**Authors:** Pashtun Shahim, Gina Norato, Ninet Sinaii, Henrik Zetterberg, Kaj Blennow, Leighton Chan, Christopher Grunseich

**Affiliations:** 1 Rehabilitation Medicine Department, National Institutes of Health (NIH) Clinical Center, Bethesda, MD 20892, USA; chanle@cc.nih.gov; 2 National Institutes of Neurological Disorders and Stroke, NIH, Bethesda, MD 20892, USA; gina.norato@nih.gov (G.N.); christopher.grunseich@nih.gov (C.G.); 3 Department of Neurology, MedStar Georgetown University Hospital, Washington, DC 20007, USA; 4 The Military Traumatic Brain Injury Initiative (MTBI2), Bethesda, MD 20814, USA; 5 The Henry M. Jackson Foundation for the Advancement of Military Medicine, Bethesda, MD 20817, USA; 6 Biostatistics and Clinical Epidemiology Service, NIH, Bethesda, MD 20892, USA; sinaiin@cc.nih.gov; 7 Department of Psychiatry and Neurochemistry, Institute of Neuroscience and Physiology, The Sahlgrenska Academy, University of Gothenburg, 431 41 Molndal, Sweden; henrik.zetterberg@clinchem.gu.se (H.Z.); kaj.blennow@neuro.gu.se (K.B.); 8 Clinical Neurochemistry Laboratory, Sahglrenska University Hospital, 431 41 Molndal, Sweden; 9 Department of Neurodegenerative Disease, UCL Institute of Neurology, Queen Square, London WC1N 3BG, UK; 10 UK Dementia Research Institute at UCL, London WC1E 6BT, UK; 11 Hong Kong Center for Neurodegenerative Diseases, Clear Water Bay, Hong Kong 518172, China; 12 Wisconsin Alzheimer’s Disease Research Center, University of Wisconsin School of Medicine and Public Health, University of Wisconsin-Madison, Madison, WI 53792, USA

**Keywords:** amyotrophic lateral sclerosis, neurofilament light, phosphorylated neurofilament heavy chain, CSF, blood

## Abstract

Background: Neurofilament proteins have been implicated to be altered in amyotrophic lateral sclerosis (ALS). The objectives of this study were to assess the diagnostic and prognostic utility of neurofilaments in ALS. Methods: Studies were conducted in electronic databases (PubMed/MEDLINE, Embase, Web of Science, and Cochrane CENTRAL) from inception to 17 August 2023, and investigated neurofilament light (NfL) or phosphorylated neurofilament heavy chain (pNfH) in ALS. The study design, enrolment criteria, neurofilament concentrations, test accuracy, relationship between neurofilaments in cerebrospinal fluid (CSF) and blood, and clinical outcome were recorded. The protocol was registered with PROSPERO, CRD42022376939. Results: Sixty studies with 8801 participants were included. Both NfL and pNfH measured in CSF showed high sensitivity and specificity in distinguishing ALS from disease mimics. Both NfL and pNfH measured in CSF correlated with their corresponding levels in blood (plasma or serum); however, there were stronger correlations between CSF NfL and blood NfL. NfL measured in blood exhibited high sensitivity and specificity in distinguishing ALS from controls. Both higher levels of NfL and pNfH either measured in blood or CSF were correlated with more severe symptoms as assessed by the ALS Functional Rating Scale Revised score and with a faster disease progression rate; however, only blood NfL levels were associated with shorter survival. Discussion: Both NfL and pNfH measured in CSF or blood show high diagnostic utility and association with ALS functional scores and disease progression, while CSF NfL correlates strongly with blood (either plasma or serum) and is also associated with survival, supporting its use in clinical diagnostics and prognosis. Future work must be conducted in a prospective manner with standardized bio-specimen collection methods and analytical platforms, further improvement in immunoassays for quantification of pNfH in blood, and the identification of cut-offs across the ALS spectrum and controls.

## 1. Introduction

Amyotrophic lateral sclerosis (ALS) is a heterogeneous neurodegenerative disorder that is characterized by progressive paralysis and weakness of the bulbar (controlling speech and swallowing), limb, and respiratory muscles [1]. Currently, there is limited effective therapy for ALS despite numerous drugs with different mechanisms that have been studied. The negative results of these trials have been attributed to pathogenetic heterogeneity in the disease as well as suboptimal trial design [2]. ALS is commonly classified as either familial or sporadic, where familial ALS accounts for approximately 10% of cases. Familial ALS has been associated with mutations in over 30 genes, but mutations in 4 genes, *C9orf72*, *SOD1* (encoding superoxide dismutase), *TARDBP* (encoding TAR DNA-binding protein 43, TDP43), and *FUS* (encoding fused in sarcoma RNA-binding protein), account for more than 70% of cases [3,4,5,6]. Proteins encoded by these genes are involved in several aspects of motor neuron biology, including DNA repair, RNA metabolism, vesicle transport, mitochondrial function, and non-cell autonomous mechanisms. Alteration of these functions can contribute to the degeneration of motor neurons in ALS.

The histopathological hallmark of ALS is progressive degeneration of the lower and upper motor neurons together with accumulation of intraneuronal protein aggregates, which in most individuals contain TDP-43 [6,7]. Several additional histopathological changes have been reported in ALS, including phosphorylated neurofilament in the degenerating motor neurons in the spinal cord and cerebral cortex of patients with ALS [8,9]. RNA-binding proteins have been reported in the aggregation of neurofilament protein, and are implicated in the pathogenesis of motor neuron disease [10]. In line with this hypothesis, reducing neurofilament levels in an *SOD1* model of ALS improved the lifespan of the animal [11].

**Figure 1:**
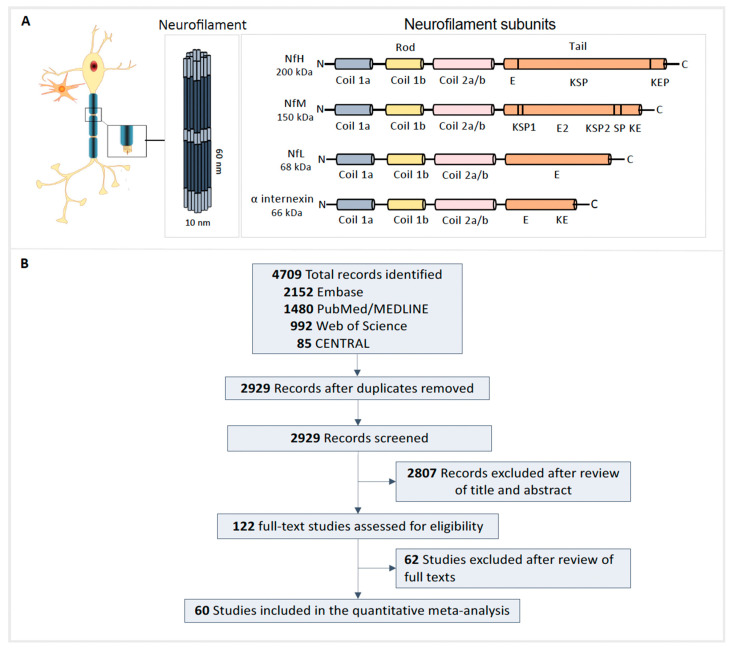
Neurofilament structures and preferred reporting items for systematic reviews and meta-analyses: flow diagram. (**A**) Structure and assembly of neurofilaments with a length of ~60 nm and a diameter of ~10 nm. Neurofilament light chain (NfL), neurofilament medium chain (NfM), neurofilament heavy chain (NfH), and α internexin are the subunits of neurofilaments. All neurofilament subunits have a conserved α-helical rod domain comprising several coiled coils, and variable amino-terminal globular head regions and carboxy-terminal globular head regions and carboxy-terminal tail domains. NfM and NfH subunits have long carboxy-terminal domains with multiple Lys-Ser-Pro repeats that are heavily phosphorylated. The tail domains of NfM and NfH radiate outward from the filament core because of the negative charges from large numbers of glutamic acid and phosphorylated serine and threonine residues. E segment, glutamic-rich segment; E1, glutamic acid-rich segment 1; E2, glutamic acid-rich segment 2; KSP, lysine–serine–proline; SP, serine–proline; KE, lysine–glutamic acid; KEP, lysine–glutamic acid–proline. (**B**) Search strategy and records identified, as well as article exclusion following screening for eligibility.

Neurofilaments are cytoskeleton proteins with a diameter of ~10 nm and are members of the intermediate filament family (Figure 1A). Neurofilaments are composed of three subunits based on their molecular mass: neurofilament light (NfL, 68–70 kDa), medium (NfM, 145–165 kDa), and heavy (NfH, 200–220 kDa) [12]. NfM and NfH are both heavily phosphorylated. NfL is abundant in the large-caliber myelinated subcortical axons that project into the deeper brain layers and the spinal cord [13]. Neurofilaments are released into the extracellular space following axonal damage and normal aging [14]. Several studies have found increased concentrations of NfL in the cerebrospinal fluid (CSF) of patients with ALS compared to controls [15,16,17,18]. Similarly, phosphorylated NfH (pNfH) measured in CSF has been shown to distinguish patients with ALS from controls with high accuracy [19]. Recent advances in immunoassay technology have made it possible to quantify NfL in blood with high analytic sensitivity, and using this new assay, blood NfL has been shown to distinguish patients with ALS from controls with high accuracy [20,21,22,23,24]. Although neurofilaments, especially NfL and pNfH, measured in CSF have been assessed as diagnostic biomarkers for ALS, their utility when measured in blood and for monitoring disease progression is yet to be examined in detail. We conducted a systematic review and meta-analysis investigating the use of neurofilaments, specifically NfL and pNfH, for diagnosis and monitoring disease progression in patients with ALS.

## 2. Methods

This study was conducted in accordance with Preferred Reporting Items for Systematic Reviews and Meta-analysis (PRISMA) reporting guidelines [25] and the Diagnostic Test Accuracy extension [26]. The review protocol with minor adjustments was registered in PROSPERO, CRD42022376939.

We performed a systematic literature search from inception to 1 August 2022, and an updated search on 17 August 2023, in the following electronic databases—PubMed/MEDLINE, Embase, Web of Sciences, and Cochrane Central Register of Controlled Trials (CENTRAL)—using Boolean operators and limiting it to English-only studies. The list of all database search strategies and results, and the study selection, are described in detail in the Supplementary Appendix. Two reviewers (P.S. and G.C.) screened titles and abstracts and full texts to assess eligibility criteria and disagreements were resolved by consensus. Overall, there was excellent agreement between the two reviewers (Cohen’s κ = 0.93).

### 2.1. Inclusion and Exclusion Criteria

We included studies that measured NfL and/or pNfH in either blood (plasma or serum), CSF, or both in patients with ALS fulfilling the El Escorial criteria [27] and various controls including those with disease mimics (e.g., spinobulbar muscular atrophy, neurologic amyotrophy, hereditary spastic paraparesis, multifocal motor neuropathy) [28]. We included peer-reviewed articles, written in English, which presented original research with human data only. We included studies that assessed (1) neurofilaments primarily in ALS, and (2) either diagnostic test accuracy, concentrations of neurofilaments, (3) the relationship between blood and CSF neurofilaments, or (4) the relationship with the functional outcome. We excluded studies that were not primarily ALS studies, or presented data in a fashion that was not compatible for extraction (e.g., figures that could not be digitized). Additional inclusion/exclusion and study selection criteria are detailed in Appendix 2 in the Supplementary Materials.

### 2.2. Outcome Assessment

Outcome assessment included the ALS Functional Rating Scale Revised (ASLFRS-R) [29].

The ALSFRS-R is used to monitor the progression of disability in patients with ALS. We included studies where the ALS disease progression rate was calculated by subtracting baseline ALSFRS-R from 48 (maximum ALSFRS-R score) and dividing by time (months) from symptom onset to the baseline (see Appendix 3 in the Supplementary Materials) [30]. Survival duration was defined as time from symptom onset to permanent assisted ventilation, tracheostomy, or death.

### 2.3. Quality Assessment

A quality assessment (P.S. and C.G.) of all included studies reporting diagnostic accuracy was conducted using the revised tool for the Quality Assessment of Diagnostic Studies 2 (QUADAS-2) [31] to assess the risk of bias in each selected study (detailed in the Supplementary Appendix).

### 2.4. Quantification of Plasma and Serum NfL

We undertook a sub-analysis of the relationship between plasma and serum NfL, and for this purpose we included 26 healthy controls (16 females and 10 males, mean age 23 years, SD 4.7) who had undergone paired plasma and serum sampling at Sahlgrenska University Hospital, Mölndal, Sweden. NfL was measured in plasma and serum using Single Molecule Array technology (Quanterix, Billerica, MA, USA) [20,21], detailed in the Supplementary Appendix.

### 2.5. Statistical Analysis

Meta-analyses of sensitivity, specificity, hierarchical summary receiver-operating characteristics curve (SROC) were performed using the R package *mada* (https://rdrr.io/rforge/mada/src/R/reitsma.R, accessed on 28 March 2024). A meta-analysis for correlation data was performed using the R package *metacor* (https://cran.r-project.org/package=metacor, accessed on 28 March 2024). Only Spearman’s rank correlations (r_s_) were used in the analysis due to the likely skewed nature of the data and since most studies reported Spearman’s rank correlations. For studies that reported other correlation methods than Spearman’s rank, the corresponding authors were contacted to provide the Spearman’s rank correlations. The relationship between the biomarkers and survival was assessed using a univariate correlation between the serum biomarkers and survival time when data were available or based on the reported hazard ratio (HR). A meta-analysis for the survival data was performed using the R package *metafor* (https://CRAN.R-project.org/package=metafor, accessed on 28 March 2024). Heterogeneity was assessed using Cochran’s *Q* test and the *I*^2^ index. Lastly, we performed sample size calculations based on (1) the pooled meta-analysis data and (2) studies with longitudinal follow-up data [32,33]. We used the n80 criteria for assessing efficiency of neurofilaments as outcome measures in future phase 2 ALS interventional trials [34]. The statistical methodology is detailed in the Supplementary Appendix. All analyses were performed using R statistical software, version 4.0.4 (R Core Team).

## 3. Results

Our search strategy identified 4709 records, and after removing duplicates, 2929 remained (Figure 1B). We excluded 2807 records after title and abstract screening. We reviewed the full text of the remaining 122 studies, of which 60 studies and 8801 participants were included in the quantitative meta-analysis (Supplementary Table S1). A list of full-text studies excluded and rationales for exclusion is provided in Supplementary Table S2.

The demographics of participants of studies included in the main analyses are presented in Table 1. The pooled median (IQR) age of onset for all ALS patients included was 61.1 (55.4–68.5) years, while the age at sampling was 62.0 (IQR 48.5–70.2) years. The pooled median disease duration from age of onset to sampling was 16.8 (IQR 11.5–31.3) months. The pooled median (ALS Functional Rating Scale Revised (ALSFRS-R)) score was 38.5 (IQR 32.1–42.3).

### 3.1. Diagnostic Accuracy of Neurofilaments

**Figure 2:**
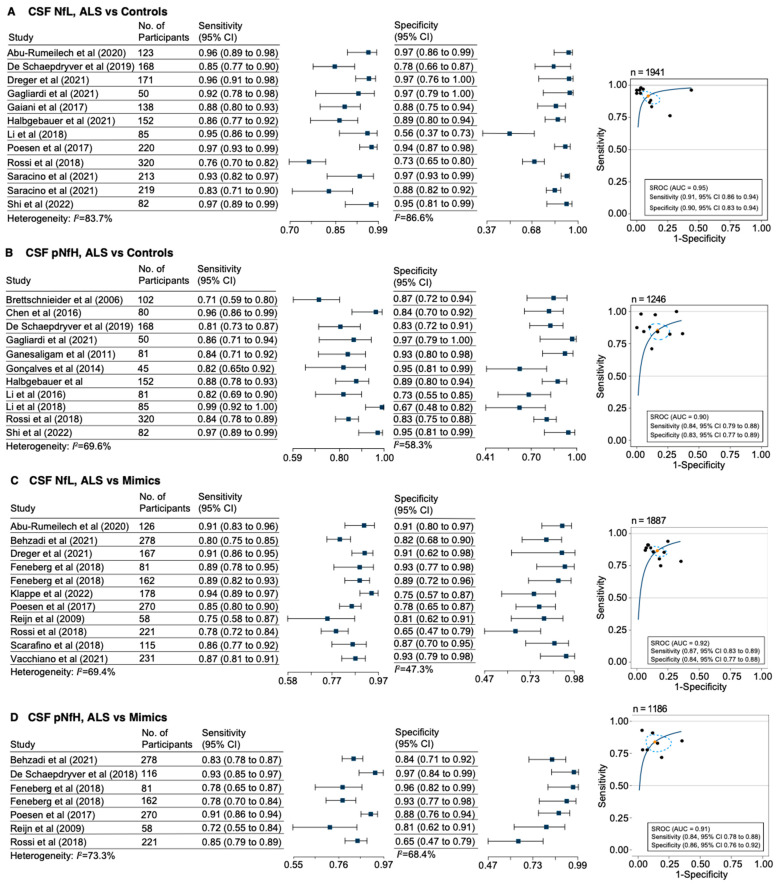
Forest plots and receiver operating characteristic curves for the diagnostic accuracy of CSF neurofilament light chain and phosphorylated neurofilament heavy chain in ALS and controls and disease mimics. Forest plots of sensitivity, specificity, and summary receiver operating characteristics (SROCs) and their confidence intervals for (**A**) NfL and (**B**) pNfH distinguishing ALS from controls. Forest plots of sensitivity and SROCs and their confidence intervals for (**C**) NfL and (**D**) pNfH distinguishing ALS from mimics are presented. Each individual dot in the SROC represents a unique study. The orange diamond represents the summary estimate of sensitivity and false-positive rate (1-specificity), and the dotted circle represents the 95% confidence region. On top of each SROC, “n” represents the total number of participants in the analyses. Feneberg et al. [17]: early symptomatic ALS vs. controls. Feneberg et al. [17]: late symptomatic ALS vs. controls. Saracino et al. [35]: *GRN* patients vs. controls. Saracino et al. [35]: *C9orf72* patients vs. controls.

The pooled mean sensitivity, specificity, and hierarchical summary receiver-operating characteristic curve of CSF NfL in distinguishing ALS patients from controls were 0.91 (95% CI, 0.86 to 0.94), 0.90 (95% CI, 0.83 to 0.94), and 0.95, respectively (*I*^2^ = 83.7%; Figure 2A). The pooled mean sensitivity, specificity, and SROC of CSF pNfH in distinguishing ALS patients from controls were 0.84 (95% CI, 0.79 to 0.88), 0.83 (95% CI, 0.77 to 0.89), and 0.90, respectively (*I*^2^ = 69.6%; Figure 2B).

The pooled mean sensitivity, specificity, and SROC curve for CSF NfL in distinguishing ALS patients from disease mimics were 0.87 (95% CI, 0.83 to 0.89), 0.84 (95% CI, 0.77 to 0.88), and 0.92, respectively (*I*^2^ = 69.4%; Figure 2C). Similarly, for CSF pNfH, the pooled mean sensitivity, specificity, and SROC curve were 0.84 (95% CI, 0.78 to 0.88), 0.86 (95% CI, 0.76 to 0.92), and 0.91, respectively (*I*^2^ = 73.3%; Figure 2D).

The pooled mean sensitivity, specificity, and SROC curve for NfL in blood were 0.92 (95% CI, 0.89 to 0.94), 0.90 (95% CI, 0.83 to 0.94), and 0.95, respectively (*I*^2^ = 55.5%; Supplementary Figure S1). The diagnostic performance of blood pNfH for ALS and controls as well as disease mimics was not assessed by meta-analysis due to the insufficient number of studies.

We also performed a meta-analysis of the continuous data (Supplementary Figure S2). These results indicate that NfL measured either in CSF, plasma, or serum distinguishes ALS from disease mimics and controls. Similar results were seen for pNfH in CSF and serum, albeit with higher variability (Supplementary Figure S2).

### 3.2. Correlation of Plasma and Serum NfL

Plasma and serum NfL concentrations were correlated (r_s_ = 0.66, 95% CI 0.35 to 0.84); however, average NfL concentrations were ~17% higher in serum compared with plasma (Supplementary Figure S3).

### 3.3. Correlation of CSF and Blood Neurofilaments

**Figure 3:**
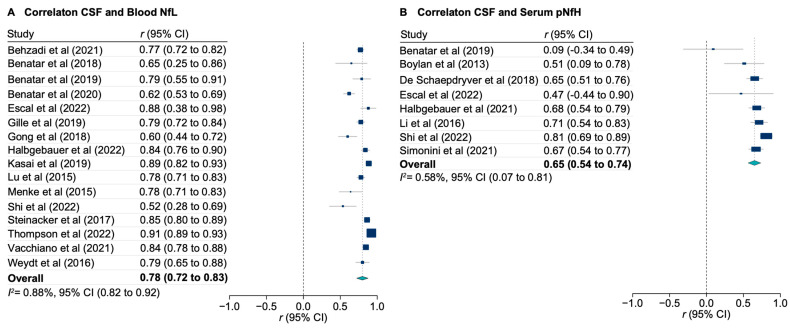
Meta-analysis of correlations between CSF and blood neurofilaments. (**A**) Correlations between cerebrospinal fluid (CSF) and blood neurofilament light (NfL). (**B**) Correlations between CSF and serum phosphorylated neurofilament heavy chain (pNfH). All correlations were calculated using the Spearman’s rank method. Markers indicate estimates, with the size of the marker indicating weight; horizontal lines represent 95% CIs; diamonds represent summary estimates, with the outer points indicating 95% CIs.

NfL measured in blood (either plasma or serum) correlated tightly with corresponding levels in CSF (pooled r_s_ = 0.78 [95% CI, 0.72 to 0.83]; Figure 3A). There was also a correlation between CSF pNfH and corresponding serum levels (pooled r_s_ = 0.65 [95% CI, 0.54 to 0.74]; Figure 3B). Due to methodological issues with quantifying pNfH in plasma, the meta-analysis of the correlations of CSF and plasma is reported separately (Supplementary Figure S4).

### 3.4. Neurofilaments and ALS Functional Rating Score, Disease Progression, and Survival

**Figure 4:**
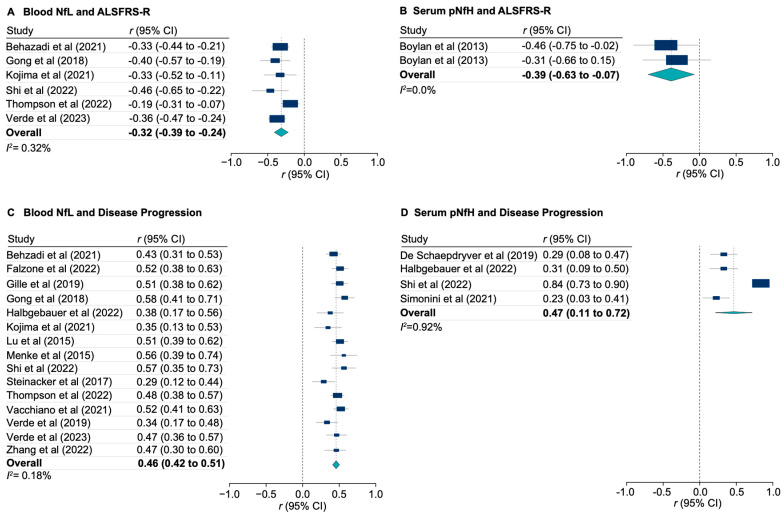
Meta-analysis of CSF and blood neurofilaments and their associations with clinical markers of ALS functional score and disease progression. Correlation of NfL (**A**) and pNfH (**B**) measured in blood with ALS Functional Rating Scale (ALSFRS-R). Correlation of NfL (**C**) and pNfH (**D**) measured in blood with disease progression. All correlations were calculated using the Spearman’s rank method. Markers indicate estimates, with the size of the marker indicating weight; horizontal lines represent 95% CIs; diamonds represent summary estimates, with the outer points indicating 95% CIs.

Higher concentrations of NfL measured in CSF or blood were associated with lower ALSFRS-R scores (pooled r_s_ = −0.35 [95% CI, −0.46 to −0.24]; *I*^2^ = 0.70%; Supplementary Figure S5A, and pooled r_s_ = −0.32 [95% CI, −0.39 to −0.24; *I*^2^ = 0.32%; Figure 4A, respectively). Similar relationships were seen for pNfH measured in CSF or serum and ALSFRS-R scores (pooled r_s_ = −0.36 [95% CI, −0.50 to −0.22]; *I*^2^ = 0.10%; Supplementary Figure S5B, and pooled r_s_ = −0.39 [95% CI, −0.63 to −0.07]; *I*^2^ = 0.0%; Figure 4B, respectively).

Also, NfL measured either in CSF or blood were associated with disease progression (pooled r_s_ = 0.44 [95% CI, 0.38 to 0.50]; *I*^2^ = 0.67%; Supplementary Figure S5C, and pooled r_s_ = 0.46 [95% CI, 0.42 to 0.51]; *I*^2^ = 0.18%; Figure 4C, respectively). Similarly, pNfH measured in either CSF or serum was associated with disease progression (pooled r_s_ = 0.41 [95% CI, 0.31 to 0.50]; *I*^2^ = 0.10%; Supplementary Figure S5D, and pooled r_s_ = 0.47 [95% CI, 0.11 to 0.72]; *I*^2^ = 0.92%; Figure 4D, respectively).

**Figure 5:**
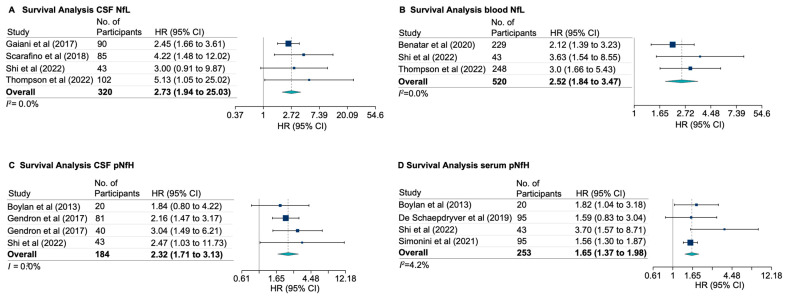
Neurofilament and Survival. (**A**) Higher CSF or blood NfL (**B**) is associated with shorter survival in patients with ALS. (**C**) pNfH measured in CSF and survival time. (**D**) pNfH measured in serum and survival time.

Pooled results of the univariate analyses showed that blood NfL correlates with survival time, where patients with higher baseline levels had shorter survival compared to those with lower levels (r_s_= –0.38 [95% CI, –0.24 to –0.51]; *I*^2^ = 0.77%; Supplementary Figure S6). Pooled results from multivariate analyses showed that higher concentrations of both CSF and blood NfL were independently associated with shorter survival (HR = 2.73 [95% CI, 1.94 to 25.03]; *I*^2^ = 0.0%, and HR = 2.52 [95% CI, 1.84 to 3.47]; *I*^2^ = 0.0%; Figure 5A,B, respectively). Similarly, higher concentrations of pNfH measured in either CSF or serum were associated with shorter survival (pooled HR = 2.32 [95% CI, 1.71 to 3.13]; *I*^2^ = 0.0%, and pooled HR = 1.65 [95% CI, 1.37 to 1.98], *I*^2^ = 4.2%]; Figure 5C,D, respectively).

### 3.5. Neurofilaments in FTD-ALS Spectrum and Genetic Forms of ALS

The studies assessing neurofilaments in the FTD-ALS spectrum are summarized in Supplementary Table S5. There were few studies that assessed neurofilaments in FTD-ALS to allow for quantitative meta-analysis. In the few studies that assessed neurofilaments across ALS and/or FTD, higher NfL was shown in patients with ALS or FTD-ALS compared to FTD alone [23,35,36,37].

The studies that assessed the performance of neurofilaments in relation to common genetic forms of the disease are summarized in Supplementary Table S6. The relationship between neurofilaments and familial forms of ALS was not assessed by meta-analysis due to the insufficient number of studies. In summary, ALS patients with *C9orf72* repeat expansion had higher CSF pNfH, faster disease progression, and shorter survival compared to those with ALS without *C9orf72* mutation [38]. In contrast, higher serum NfL and not serum pNfH levels were associated with the presence of *C9orf72* repeat expansion, and higher baseline levels of serum NfL were independently associated with shorter survival [24]. Also, higher plasma NfL levels were found in ALS patients with *GRN* mutations compared to those with *C9orf72* [35,37].

### 3.6. Risk of Bias and Concerns for Applicability Assessment

The results from the Quality Assessment of Diagnostic Studies 2 (QUADAS-2) which assess the risk of bias and concerns of applicability are summarized in Supplementary Figure S7. Overall, the risk of bias and applicability concerns was low, except for the patient selection bias.

### 3.7. Neurofilaments as Outcome Measures in Future Clinical Trials

**Figure 6:**
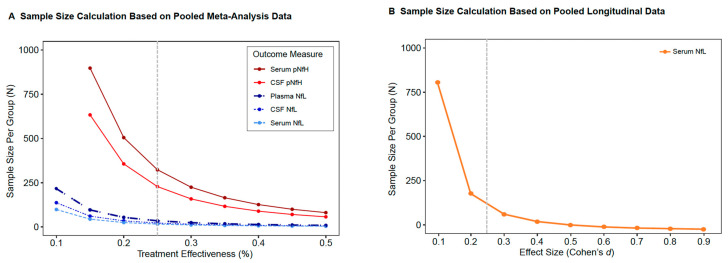
Clinical trial sample size requirements based on neurofilaments. (**A**,**B**) Sample size requirements for a placebo-controlled clinical trial of a treatment aimed at reducing neurofilament levels in cases relative to controls. Sample sizes are plotted against potential treatment effectiveness (**A**) or effect size (Cohen’s *d*) estimates (**B**). The gray dashed vertical line indicates the 25% effectiveness level used in trials of drug intervention in other neurodegenerative diseases [34].

We performed power calculations based on (*1*) the pooled meta-analysis data and (*2*) studies with longitudinal follow-up data. We compared the sample sizes needed to detect intervention effects using NfL and pNfH measured in various fluid sources (plasma, serum, or CSF neurofilaments), as applicable. The relationship between treatment effects (e.g., assumption for the treatment effectiveness (%) in slowing neuronal loss relative to controls) and sample size for different biomarkers and fluid sources functioning as outcome measures is summarized in Figure 6A. Sample size estimates using longitudinal follow-up data based on expected changes in serum NfL over time are shown in Figure 6B. For an interventional trial of ALS, including CSF, plasma, or serum NfL, sample sizes of 25, 35, and 16 are required, respectively, per arm with a treatment effect of 25%. In comparison, using CSF or serum pNfH results in sample sizes of 228 and 323, respectively, per arm with a treatment effect of 25%. Using longitudinal data, a sample size of 36 would be needed for a moderate effect size of 0.5 in paired designs of treatment effectiveness (Figure 6B). A detailed relationship of the effect estimates and sample size is provided in the Supplementary Appendix (Tables S3 and S4).

## 4. Discussion

The results of the meta-analyses presented in this paper show that both NfL and pNfH measured in CSF have high diagnostic accuracy in distinguishing patients with ALS from controls and disease mimics. Furthermore, blood NfL is highly correlated with CSF NfL in ALS. Importantly, higher NfL and pNfH measured in either CSF or blood correlated with decreased ALS functional scores and increased rate of disease progression, while higher CSF or blood NfL levels were also associated with shorter survival. Lastly, the meta-analysis data indicate that NfL may be a more sensitive biomarker than pNfH for future interventional trials.

CSF NfL is a biomarker of subcortical axonal degeneration and has been assessed as a biomarker for acute brain injury, including traumatic brain injury [39] and brain hypoxia [27], as well as for several neurodegenerative diseases, including ALS [40]. As expected, the results of this analysis suggest that NfL measured in CSF or blood could distinguish ALS patients from controls and disease mimics to improve diagnostic accuracy. Importantly, NfL measured in either CSF or blood correlated with ALS functional outcome scores, disease progression rate, and survival. Together, these results further support the potential utility of NfL as a clinically diagnostic and prognostic biomarker for ALS.

NfH undergoes post-translation phosphorylation, which facilitates neurofilament transport along axons and regulates axonal stability and protein–protein interactions [41]. CSF pNfH has been assessed extensively in patients with ALS, where CSF pNfH could distinguish patients with ALS from controls and disease mimics [38,42,43,44,45,46]. In direct comparison, the results of the meta-analysis confirm the diagnostic utility of CSF pNfH in ALS. A few studies have also measured pNfH in blood [24,47,48]. However, there are limitations of measuring pNfH in blood, including matrix effects involving analyte aggregation that may contribute to plasma NfH variability [49,50].

We also assessed NfL in paired plasma and serum samples of healthy controls and found a strong correlation between plasma and serum and that NfL concentrations are ~17% higher in serum than plasma. In comparison, a previous study [51] measuring NfL in samples of patients with various neurologic conditions found that the NfL level is between 10 and 12% higher in serum than in plasma. These results indicate that plasma and serum levels are not interchangeable within the same study.

The findings that CSF NfL correlated with blood NfL in patients with ALS is consistent with the results of previous studies of patients with other neurodegenerative diseases and healthy controls [21,52]. In comparison, the correlation of CSF and serum pNfH was variable and not as strong as the association between CSF and blood NfL, which could be due to the methodological difficulty of accurately quantifying pNfH in serum as serum pNfH is sequestered in hetero-aggregate immune complexes [53]. Another plausible explanation could be the energy-saving adaptive response to neurodegeneration in ALS, where there is a shift in protein expression from larger NfH to smaller NfL, resulting in a shift in the conserved stoichiometry of neurofilament isoforms [54]. Together, these results suggest that blood NfL reflects CSF NfL, and blood NfL (either serum or plasma) may be used instead of CSF during both routine testing and interventional trials of ALS. Further comparing the performance of NfL and pNfH, our results suggest that CSF NfL and pNfH perform equally well in distinguishing ALS patients from controls as well as disease mimics [55]. However, when these biomarkers are measured in blood, NfL performs better than pNfH, especially blood NfL, which shows stronger associations with survival [24]. There are several plausible reasons as to why NfL measured in blood performs better than pNfH, including that ultrasensitive immunoassays to measure NfL in either plasma or serum have been optimized to reduce matrix effects [24,56], while there is still variability in immunoassays measuring pNfH in blood [49]. Furthermore, pNfH measured in longitudinal plasma samples from patients with ALS decreased with disease progression, while NfL maintained a steady trajectory with disease progression [49]. This could be due to a shift in the highly conserved stoichiometry of neurofilament isoforms in ALS, as discussed earlier; however, further longitudinal studies of pNfH using ultrasensitive immunoassays are needed to address this issue [54]. In a clinical trial, a reduction in the bioavailability of pNfH with disease progression may make the interpretation of the treatment effects difficult.

Lastly, to assess the potential utility of neurofilaments as pharmacodynamic biomarkers in future phase 2 trials of ALS, we performed a sample size calculation using the meta-analysis data. These results show that NfL measured either in blood (serum or plasma) or CSF performed essentially equally well as a sensitive pharmacodynamic marker and is a more sensitive outcome measure as compared to pNfH. The results of such power calculations, although powerful based on the large sample size, should be interpreted with caution due to the heterogeneity between studies and within the disease itself. The rationale for this type of sample size calculation was to assess the feasibility of potentially incorporating neurofilaments in future trials and to compare the sensitivity of NfL and pNfH as outcome measures. Our results are comparable to the sample size calculation of a previous study [24] showing that serum NfL is a more sensitive outcome measure compared to pNfH in future phase 2 trials of ALS.

We present novel and clinically relevant data related to SROC curves, the relationship between neurofilaments and the ALS functional score, clinical disease progression measures, survival, the correlation between CSF and blood neurofilaments, the correlation between plasma and serum, mean and median concentrations for all included patients and subgroups, and effect sizes for various treatment effects. However, our study is not without limitations. First, we found evidence of moderate heterogeneity for NfL and pNfH measured in CSF and in studies reporting diagnostic utility, while we found no evidence of heterogeneity for the analysis with disease progression and survival. This could be due to the high variability in early immunoassays for detecting NfL and pNfH in CSF. Second, we could not perform a meta-analysis on whether neurofilaments could distinguish ALS from FTD due to the limited number of studies; however, patients with ALS or FTD-ALS had higher serum NfL compared to those with FTD alone [23,35,36,37]. Third, few studies have assessed the utility of neurofilaments across various genetic forms of ALS. This limited number of familial ALS studies indicates that ALS patients with *C9orf72* repeat expansion have higher CSF pNfH, faster disease progression, and shorter survival compared to those with ALS without *C9orf72* mutation [38]. When NfL and pNfH were measured in serum, only higher levels of serum NfL were associated with the presence of *C9orf72* repeat expansion and shorter survival [24]. Fourth, few studies have assessed neurofilaments in pre-symptomatic ALS, which is important for detection and prediction of when manifest disease is likely to emerge. Only a few longitudinal studies have found that neurofilaments, especially NfL measured in either blood or CSF or CSF pNfH (not blood), are elevated as far back as a year before the emergence of the earliest clinical symptoms (Supplementary Table S7). Efforts are underway to evaluate plasma NfL as a pharmacodynamic biomarker in pre-symptomatic familial ALS trials [57]. Lastly, there is a lack of consensus for the cut-off for NfL and pNfH in any of the sources for ALS.

In conclusion, both NfL and pNfH measured in CSF or blood show high diagnostic utility and association with ALS functional scores and disease progression, while CSF NfL correlates strongly with blood (either plasma or serum) and is also associated with survival, supporting its use in clinical diagnostics and prognosis. Furthermore, blood NfL may have value as a pharmacodynamic biomarker and should be incorporated in phase 2 trials. Future work must be conducted in a prospective manner with standardized bio-specimen collection methods and analytical platforms, further improvement in immunoassays for quantification of pNfH in serum, and the identification of cut-offs across the ALS spectrum and in controls.

## Figures and Tables

**Table 1:** Demographic and clinical data for all included studies in the quantitative meta-analysis ^a^.

Variable	All Cohorts	ALS	Disease Mimics ^b^	Controls
Included studies, no.	60	60	16	44
Study participants				
Total no.	8801	6064	539	2198
Men	4529	3306	286	950
Women	3148	2106	142	906
Missing, sex	330	190	26	56
Age of onset, median (IQR)	–	61.1 (55.4–68.5)	–	–
Age at sampling, median (IQR)	59.6 (47.6–69.3)	62.0 (48.5–70.2)	62.6 (54.2–74.1)	55.3 (44.5–66.8)
Disease duration from onset to sampling, median (IQR), months	–	16.8 (11.5–31.3)	–	–
ALSFRS-R, mean (SD)	–	37.4 (6.2)	–	–
ALSFRS-R, median (IQR)	–	38.5 (32.1–42.3)	–	–

Abbreviation: ALSFRS-R, Amyotrophic Lateral Sclerosis Functional Rating Scale Revised. ^a^ Not every study reported demographic data or ALSFRS-R scores; therefore, the values are based on the total sample size of the studies that reported on each specific demographic factor. Also, several studies included additional neurologic controls which are not included in this table. ^b^ Disease mimics (e.g., spinobulbar muscular atrophy, neurologic amyotrophy, hereditary spastic paraparesis, multifocal motor neuropathy).

## Supplementary Materials

The following supporting information can be downloaded at https://www.mdpi.com/article/10.3390/genes15040496/s1.
