# Supplementary material for: Neurofilaments in Sporadic and Familial Amyotrophic Lateral Sclerosis: A Systematic Review and Meta-Analysis
Source: Genes (Basel). 2024 Apr 16;15(4):496. doi: 10.3390/genes15040496 (PMC11050235; doi:10.3390/genes15040496)
Supplement: Supplementary file 1 [file genes-15-00496-s001.zip › genes-2922331-supplementary.pdf]

Supplementary Appendix

Shahim P, Norato G, et al. Neurofilaments in amyotrophic lateral sclerosis: a system review and meta-analysis.

Appendix 1. Detailed Search Methods.
Appendix 2. Data Inclusion for Studies with Duplicated Data
Appendix 3. Outcome Assessment.
Appendix 4. QUADAS-2.
Appendix 5. Quantification of Plasma and Serum NfL.
Appendix 6. Statistical Analysis.
Table S1. Study Characteristics.
Table S2. Excluded Studies.
Figure S1. Forest Plots and Receiver Operating Characteristics Curves for the Diagnostic Accuracy of Serum NfL.
Figure S2. Group Differences in Biomarkers Between ALS, ALS Mimic, and Controls.
Figure S3. Correlation Plasma and Serum NfL.
Figure S4. Meta-analysis of Correlation CSF and Plasma pNfH.
Figure S5. Meta-analysis of Relationships of pNfH and NfL Measured in CSF and ALSFRS-R and Disease Progression.
Figure S6. Meta-analysis of Correlation Between Serum NfL and Survival Time.
Figure S7. Risk of Bias and Concerns for Applicability Assessment
Table S3. Sample Size Per Group for Various Effect Estimates.
Table S4. Sample Size for Paired Assessments at Various Effect Sizes using Longitudinal Studies.
Table S5. Review of Studies of Neurofilaments Across ALS-FTD Spectrum.
Table S6. Review of Studies of Neurofilaments in ALS Mutation Carriers.
Table S7. Review of Studies of Neurofilaments in Presymptomatic ALS patients.

References.

Appendix 1. Detailed Search Methods.

Literature Search

This study was conducted in accordance with Preferred Reporting Items for Systemic Reviews and Meta-analysis (PRISMA) reporting guidelines[1] and the Diagnostic Test Accuracy extension.[2] The review protocol with minor adjustments was registered in [3-37]
We performed a systemic literature search from inception to August 1, 2022, and an updated search on August 17, 2023: PubMed/MEDLINE, Embase, Web of Sciences, and Cochrane Central Register of Controlled Trials (CENTRAL). Our search included the following search terms: biomarker, neurofilament light, NfL, NF-L, phosphorylated neurofilament heavy chain, pNfH, NF-H AND amyotrophic lateral sclerosis, and ALS. In addition to the database searches, we reviewed the reference lists of all included articles for potential eligibility. The database search was conducted by a National Institutes of Health Librarian/informationist.

Study Selection

After the completion of all databases searches, all results were imported into citation management software EndNote 20 (Clarivate Analytics) and duplicates were removed. Next, the citations were exported into Covidence systemic review screening software (Veritas Health Innovations, Ltd.), Using Covidence, two reveiwers (P.S. and G.C.) screened title and abstract to assess eligibility criteria and disagreements were resolved by consensus. Full text screening was conducted by (P.S. and G.C.) independently. Overall, there was an excellent agreement between the two revieweers (Cohen’s $\kappa = 0.93$).

Additional Exclusion Criteria

We excluded conference proceedings and studies where we could not extract the data (e.g., figures could not be digitized), as well as studies where there was uncertainty regarding the demographic factors or biomarker concentration units.

Appendix 2. Data Inclusion for Studies with Duplicated Data.

Data that were duplicated across more than one publication were not duplicated in our analysis nor included in our summary data in Table 1. However, duplicated data that reported their data in a different fashion, e.g., ROC curves or summary statistics versus association with disease progression may have contributed to different aspects of this meta-analysis. Publications with duplicated data are listed below:

Kojima *et al.,* 2021. *PLOS ONE:* overlap with Kasai et al., 2019.

- Included the correlation between CSF NfL and ALSFRS-R score and disease progression.

Appendix 3. Outcome Assessment.

The ASLFRS-R is a validated scale that measures physical function in carrying out activities of daily living in patients with ALS.[38] We extracted ALSFRS-R for assessing the relationship between functional outcome and neurofilaments. The ALSFRS-R is also used to monitor the progression of disability in patients with ALS. We included those studies where the ALS disease progression rate was calculated by subtracting baseline ALSFRS-R from 48 (maximum ALSFRS-R score), divided by time (months) from symptom onset to baseline.[39] This is a commonly reported estimate of disease progression. Studies that used the D50 disease progression model instead of ALSFRS-R were excluded from the meta-analysis of neurofilaments and disease progression. Publications using the D50 method[40] instead of ALSFRS-R are outlined below:

Dreger *et al.,* 2021. *Frontiers in Neuroscience.*

Poesen *et al.,* 2017. *Neurology*:

- Modeled disease progression using the D50 and ALSFRS-R score.

Appendix 4. QUADAS-2.

A quality assessment of all included studies was conducted using the revised tool for the Quality Assessment of Diagnostic Studies 2 (QUADAS-2) [41] to assess the risk of bias in each selected study. Two investigators (P.S. and C.G.) assessed all articles independently and discrepancies were resolved by consensus. The QUADAS-2 tool evaluated four bias categories: patient selection, index test, reference test, and flow of timing of testing. It also grades the risk that this bias decreases the applicability of the results in the target population. Studies that were considered at high risk of bias were not included in the analysis. Studies that did not report diagnostic test accuracy or the data was not available the QUADAS-2 was not applied.

Appendix 5. Quantification of Plasma and Serum NfL.

We undertook a subanalysis of the relationship between plasma and serum NfL and for this purpose we included 26 healthy controls (16 females and 10 males, mean age 23 years, SD 4.7) who had undergone paired plasma and serum sampling at Sahlgrenska University Hospital, Mölndal, Sweden. All participants provided written informed consent. Blood samples were collected by venipuncture into EDTA or serum tubes and centrifuged within 20-60 minutes. Plasma and serum samples were aliquoted and stored at −80°C pending biochemical analysis. NfL was measured in plasma and serum using the Single Molecule Array technology (Quanterix, Billerica, MA, USA).[42, 43] All analytes had an average coefficient of variance <5%. The comparison of NfL in plasma and serum has not been published previously.

Appendix 6. Statistical Analysis.

The statistical plan was developed together with two statisticians (G.N. and N.S.). For meta-analysis of diagnostic accuracy, we extracted area under the curve (AUC), sensitivity and specificity. Forest plots of sensitivity and specificity was performed. Meta-analyses for AUC were performed using R package *mada* (https://rdrr.io/rforge/mada/src/R/reitsma.R). For studies that reported AUC and not the sensitivity and specificity, authors were contacted to provide the sensitivity and specificity. For estimating a hierarchical summary receiver-operating characteristics curve, a bivariate modeling approach in *mada* was taken on the recommendation of the authors. Median concentrations and spread (interquartile range or range) and or mean (standard deviation) for each biomarker were extracted for available studies. We also performed

a meta-analysis of medians for each biomarker using the quantile estimation method (*metamedian* package in R statistical software).[44] Meta-analysis for correlation data was performed using R package *metacor* (https://cran.r-project.org/package=metacor). Only Spearman's rank correlations (r_s) were used in the analysis due to the likely skewed nature of the data and since most studies reported Spearman's rank correlations. For studies that reported other correlation methods than Spearman, the corresponding authors were contacted to provide the Spearman correlations. In addition, we summarized overall study characteristics stratified by disease and controls. We pooled mean age using a weighted fixed effects model (*meta* package in R statistical software). In studies that reported age as median (IQR), the mean (SD) was estimated.[45] The variability in studies and/or patients for each comparison is a reflection of difference in data collection and reporting between studies; some studies reported ROC curve data and some studies reported group summary statistics as median (IQR) or mean (SD). For studies that reported log-transformed means, authors were contacted to provide median (IQR) and mean (SD). The relationship between the biomarkers and survival was assessed either using a univariate correlation (Spearman's rank correlation) between the serum biomarkers and survival time when data was available or based on reported hazard ratio (HR). Meta-analysis for survival data was performed using R package *metafor* (https://CRAN.R-project.org/package=metafor). Heterogeneity was assessed using the Cochran's Q test and the I^2 index. Lastly, we performed sample size calculations two ways: (*1*) based on the pooled meta-analysis data, and (*2*) based on two studies with longitudinal follow-up data. As per Witzel *et al.*, NfL was demonstrated as an important prognostic biomarker for sample size estimations that reliably compensated for clinical heterogeneity. Further, Benatar et al. supported the preferential use of serum NfL over pNfH for implementation in future trials. To be thorough, we thus compared the sample sizes needed to detect intervention effects using NfL and pNfH measured in various fluid sources (plasma, serum, or CSF neurofilaments). The longitudinal data only used serum NfL; holding variance constant, sample sizes were estimated based on paired comparisons of expected change in NfL at ranging effect sizes. Effect sizes were calculated using Cohen's *d*.[46] We used the n80 criteria for assessing the efficiency of neurofilaments as outcome measures in future phase 2 ALS interventional trials.[47] The required sample size per calculation method assumed 80% power to detect a significant effect ($\alpha = 0.05$) and varied as a function of putative

treatment effectiveness or effect size. All analyses were performed using R statistical software, version 4.0.4 (R Core Team).

**Table S1. Study Characteristics**						
**Study**	**Study Design**	**Study Cohorts**	**Country**	**Biomarkers Assessed**	**Assay**	**Number of Participants**
Abu-Rumeileh et al (2020)[48] PMID: 32100123	Prospective	ALS, ALS mimics, and healthy controls	Italy	CSF NfL	ELISA	169
Behzadi et al (2021)[49] PMID: 34764380	Retrospective	ALS, ALS mimics, and controls	Sweden	CSF pNfH, CSF NfL, and plasma NfL	ELISA and Simoa	287
Benatar et al (2018)[7] PMID: 30014505	Prospective	Pre-symptomatic familial ALS and controls	USA	CSF and serum NfL	MSD	145
Benatar et al (2019)[3] PMID: 31432691	Prospective	Pre-symptomatic familial ALS and controls	USA	CSF pNfH, serum pNfH, CSF NfL, serum NfL	ELISA	115
Benatar et al (2020)[4] PMID: 32385188	Prospective	ALS, primary lateral sclerosis, and progressive muscle atrophy	USA	CSF pNfH, serum pNfH, CSF NfL, and serum NfL	Simoa	260
Boylan et al (2012)[5] PMID: 23117489	Prospective	ALS	USA	CSF, plasma, and serum pNfH	ELISA	63
Brettschneider et al (2006)[6] PMID: 16567701	Prospective	ALS, Alzheimer's disease, and healthy controls	Germany	CSF pNfH	ELISA	175
Costa et al (2021)[50] PMID: 34359293	-	ALS and other neurological disroders	Portugal	CSF pNfH	ELISA	58

Chen et al (2016)[51] PMID: 27634542	Prospective	ALS and controls	China	CSF pNfH	ELISA	80
De Schaepdryver et al (2018)[52] PMID: 29054919	Retrospective	ALS, disease controls, and ALS mimics	Belgium	CSF and serum pNfH	ELISA	331
De Schaepdryver et al (2019)[53] PMID: 31518073	Retrospective	ALS, mild cognitive impairment, and controls	Belgium	Serum pNfH	ELISA	215
De Schaepdryver et al (2020)[54] PMID: 32029541	Retrospective	ALS	Belgium and Italy	Serum	ELISA	383
De Schaepdryver et al (2023)[55] PMID: 36047371	Retrospective	ALS, disease control	Belgium	CSF NfL and CSF pNfH	ELISA	348
Dreger et al (2021)[9] PMID: 36047371	Prospective	ALS	Germany	CSF NfL	ELISA	238
Escal et al. 2022[10] PMID: 34313819	Retrospective	ALS, FTD-ALS, FTD, and PPD	France	CSF and plasma NfL and pNfH	Simoa	81
Falzone et al. 2020[11] PMID: 32306171	Retrospective	ALS	Italy	Serum pNfH	ELISA	219
Falzone et al. 2022[12] PMID: 35263489	Prospective	ALS, ALS mimics, other neurodegenerative disorders, and controls	Italy	Serum NfL	Simoa	328

Feneberg et al. 2018[56] PMID: 29212830	Prospective	Early and late ALS, other neurologic diseases, and motor neuron disease mimics	Germany	CSF NfL and pNfH and serum NfL	ELISA and Simoa	253
Gagliardi et al. 2021[13] PMID: 33609080	Prospective	ALS, spinal muscle atrophy and healthy controls	Italy	CSF NfL and CSF pNfH	ELISA	129
Gaiani et al. 2017[14] PMID: 28264096	Retrospective	ALS, FTD, motor neuron disease and controls	Italy	CSF NfL	ELISA	176
Ganesalingam et al. 2011[16] PMID: 21418221	Retrospective	ALS, disease control and healthy control	USA	CSF pNfH	ELISA	163
Ganesalingam et al. 2013[15] PMID: 23134506	Retrospective	ALS and disease controls	Sweden	CSF pNfH	ELISA	290
Gendron et al. 2017[57] PMID: 28628244	Prospective	*C9ORF72*-ALS versus non-*C9ORF72*-ALS	USA/Multi-site	CSF pNfH	ELISA	242
Gille et al. 2019[58] PMID: 29908069	Prospective	ALS, ALS mimics, and disease controls	Belgium	CSF and serum NfL	ELISA	250
Gong et al. 2018[17] PMID: 29898446	Prospective	ALS and controls	China	CSF and serum NfL	ELISA	120
Gonçalves et al (2015)[18] PMID: 25261856	-	ALS	Portugal	CSF pNfH	ELISA	46

Halbgebauer et al (2021)[59] PMID: 34417339	Prospective	ALS, FTD, AD, PD, PDD, CJD, and non-neurodegenerative controls	Germany	CSF NfL, serum NfL, CSF pNfH, and serum pNfH	ELISA	294
Illan-Gala et al. 2018[60] PMID: 30291183	Prospective	ALS, FTD, and controls	Spain	CSF NfL	ELISA	173
Kasai et al. 2019[61] PMID: 31742901	Prospective	ALS and non-neurodegenerative controls	Japan	CSF and plasma NfL	Simoa	150
Kojima et al. 2021[20] PMID: 34843548	Retrospective	ALS	Japan	CSF and plasma NfL	Simoa	75
Kläppe et al. 2019[19] PMID: 34151677	Retrospective	ALS, ALS mimics, and healthy controls	Sweden	CSF NfL	ELISA	286
Li et al. 2016[22] PMID: 27423602	Prospective	ALS, multiple system atrophy, and controls	China	CSF and plasma pNfH	ELISA	93
Li et al (2018)[21] PMID: 30210445	Prospective	ALS and controls	China	CSF NfL and CSF pNfH	ELISA	86
Lu et al (2015)[23] PMID: 25934855	Prospective	ALS and healthy controls	England	CSF and blood NfL	ELISA	245
Menke et al (2015)[62] PMID: 26273687	-	ALS and controls	England	CSF NfL	ELISA	42
Poesen et al (2017)[25] PMID: 28500227	Prospective	ALS, neurologic disease controls, and disease mimics	Belgium	CSF NfL and CSF pNfH	ELISA	586

Reijn et al (2009)[26] PMID: 19296046	Retrospective	ALS and ALS mimics	Netherlands	CSF NfL and CSF pNfH	ELISA	58
Rosengren et al (1996)[63] PMID: 8863508	Retrospective	ALS, neurologically healthy controls, and other neurodegenerative diseases	Sweden	CSF NfL	ELISA	92
Rossi et al. 2018[64] PMID: 29322259	Prospective	ALS and mixed neurologic disease	Italy	CSF NfL and pNfH	ELISA	320
Saracino et al. 2021[65] PMID: 34349004	Prospective	*C9ORF72*-ALS and *GRN* cohorts, and controls	France	Plasma NfL	Simoa	352
Scarafino et al. 2018[66] PMID: 30116940	Retrospective	ALS, disease mimics, and non-neurodegenerative diseases	Italy	CSF NfL	ELISA	166
Schreiber et al. 2018[67] PMID: 30187162	Retrospective	ALS and disease controls	Germany	CSF NfL	ELISA	122
Shi et al. 2021[27] PMID: 34866307	Prospective	ALS and noninflammatory neurologic controls	China	CSF NfL, serum NfL, CSF pNfH, and serum pNfH	ELISA	82
Simonini et al. 2021[28] PMID: 34829852	Retrospective	ALS, primary lateral sclerosis,	Switzerland	CSF and serum pNfH	ELISA	143

Steinacker et al. 2017[30] PMID: 27819158	Prospective	ALS and controls	Germany	CSF and serum NfL	ELISA	153
Sugimoto et al. 2020[35] PMID: 33424740	Prospective	ALS and healthy controls	China	Serum NfL	Simoa	50
Sun et al. 2020[31] PMID: 32982935	Prospective	ALS, other neurological disorders and controls	China	CSF NfL	ELISA	117
Thompson et al. 2019[32] PMID: 31123140	Prospective	ALS, PLS, disease mimics, and controls	England	CSF pNfH	ELISA	134
Thompson et al. 2022[33] PMID: 35224491	Prospective	ALS, disease controls, and healthy controls	England	CSF and plasma NfL	ELISA	439
Tortelli et al. 2012[68] PMID: 22680408	Prospective	ALS, chronic inflammatory demyelinating neuropathy, and other neurodegenerative diseases	Italy	CSF NfL	ELISA	83
Thouvenot et al. 2020[34] PMID: 31437330	Prospective	ALS and controls	France	Serum NfL	Simoa	198

Vacchiano et al. 2021[69] PMID: 34744694	Prospective	ALS and disease mimics	Italy	CSF and plasma NfL	Simoa	231
Verde et al. 2019[70] PMID: 30309882	Prospective	ALS, other neurodegenerative diseases, and without neurodegenerative diseases	Germany	Serum NfL	Simoa	283
Verde et al. 2023[71] PMID: 37009451	Retrospective	ALS and healthy control	Italy	Serum NfL	Simoa	255
Weydt et al. 2016[36] PMID: 26528863	Prospective	Symptomatic and asymptomatic ALS carriers	Germany and Sweden	CSF NfL, serum NfL, and CSF pNfH	ELISA	95
Witzel et al. 2021[72] PMID: 34433481	Prospective	ALS	Multisite	Serum NfL	Simoa	125
Yamada et al. 2021[73] PMID: 33737450	Prospective	ALS and controls	Japan	Serum NfL	Simoa	113
Zecca et al. 2022[37] PMID: 35715961	Retrospective	ALS and controls	Italy	Plasma pNfH	ELISA	256
Zetterberg et al. 2007[74] PMID: 17903209	Retrospective	Sporadic ALS, familial ALS, other neurological disorder, and healthy controls	Sweden	CSF NfL	ELISA	325
Zhang et al. 2022[75] PMID:	Prospective	ALS	China	Serum NfL	Simoa	103

35280276						

Table S2. Excluded Studies							
**Study**	**Study Design**	**Study Cohorts**	**Country**	**Biomarkers Assessed**	**Assay**	**Total *N***	**Reason for Exclusion**
Bjornevik et al (2021)[8] PMID: 34380747	Prospective	ALS and controls	USA	Plasma NfL	Simoa	271	Survival analyses and data could not be extracted from the figures
Brodovitch et al (2021)[76] PMID: 33436881	Prospective	ALS and peripheral inflammatory polyneuropathy	France	CSF and serum NfL	ELISA	154	Demographic data could not be extracted for all the groups
Delaby et al. (2020)[77] PMID: 32514050	Retrospective	Down syndrome, Alzheimer's dementia, dementia with Lewy Bodies, Frontotemporal dementia, corticobasal syndrome, progressive supranuclear palsy, and cognitively normal controls	Spain	CSF NfL	ELISA	535	Not a primary ALS study
Davies et al (2023) PMID: 37292457	Prospective	ALS and primary lateral sclerosis and 19 alternative diagnoses	UK	Serum NfL	ELISA	133	Teritiary clinic referral for suspect ALS
Dorst et al (2023) PMID: 36917918	Prospective	ALS mutations carriers and non-carriers	Germany and Sweden	Body composition and Serum NfL	ELISA	133	Not primarily an ALS diagnostic or prognostic study assessing neurofilaments

Gray et al. (2020)[78] PMID: 32558597	Retrospective	ALS and controls	Germany	CSF NfL, plasma NfL, serum NfL, CSF pNfH, plasma pNfH, and serum pNfH	Multiple Assays	10	Assay reliability and inter-laboratory variability
Huang et al. (2020)[79] PMID: 32515902	Retrospective	ALS and controls	USA	CSF NfL, plasma NfL, CSF pNfH, plasma pNfH	ELISA (MSD, Simoa)	149	Reported percent changes and values from figures could not be accurately digitized
Ingannato et al (2021)[80] PMID: 34539331	Prospective	ALS and bv-FTD and nfv-PPA	Italy	Plasma NfL	Simoa	106	Data log-transformed and could not be accurately converted
Kaiserova et al (2017)[81] PMID: 28185258	Prospective	ALS and controls	Czech Republic	CSF pNfH	ELISA	31	Neurofilament values could not be accurately extracted from the figures
Lombardi et al (2019)[82] PMID: 30787165	Prospective	ALS, SBMA, and controls	UK and Italy	Plasma NfL, serum NfL	Simoa	292	Not primarily an ALS study and neurofilament values could not be accurately extracted from the figures
Lu et al (2014)[24] PMID: 25009280	Prospective	ALS and controls	England	Plasma pNfH	ELISA	240	Data could not be extracted from the figures
Li et al (2023) PMID: 37070132	Prospective	ALS and spinal muscle atrophy	China	Serum Cr, CSF NfL, and CSF pNfH	ELISA		Not primarily and ALS study

Meyer et al (2023) PMID: 36899448	Prospective	ALS	Germany	Serum NfL	Simoa	1378	Data z transformed
McCombe et al. 2014[83] PMID: 25958264	Prospective	ALS and controls	Australia	Serum pNfH	ELISA	157	Data could not be extracted from the figures
Oeckl et al. 2016[84] PMID: 27415180	Retrsopective	ALS and controls	Multisite	CSF NfL, CSF pNfH	ELISA	150	Assessed neurofilament variability between centers
Pawlitzki et al. 2018[85] PMID: 30631300	Retrospective	ALS, Primary progressive multiple sclerosis, and healthy controls	Germany	CSF NfL	ELISA	150	Primarily a multiple sclerosis study that included an ALS group for comparison
Sabbatini et al. 2021[86] PMID: 34264016	Retrospective	ALS and other MNDs and controls	Italy	CSF NfL, serum NfL	ELISA	160	Not primarily an ALS study.
Steinacker et al. 2015[29] PMID: 26296871	Prospective	ALS, primary lateral sclerosis, motor neuron disease mimics and neurological control groups	Germany	CSF NfL and pNfH	ELISA	455	Not primarily an ALS study. Neurofilaments assessed across many MNDs including ALS
Tortelli et al. 2014[87] PMID: 24750431	Prospective	Sporadic ALS	Italy	CSF NfL	ELISA	37	Data on NfL could not be extracted from the figures
Verde et al. 2023	Restropective	ALS	Italy	Plasma P-tau 181	ELISA	29	P-tau study

PMID: 37369876							
Yamada et al. 2021[73] PMID: 33737450	Prospective	ALS and controls	Japan	Serum NfL	Simoa	113	No primarily an ALS study and sensitivity, specificity and IQR could not be extracted
Wilke et al. 2018[88] PMID: 30009206	Prospective	Hereditary spastic paraplegia, ALS, and controls	Germany	Serum NfL	Simoa	225	Primarily a hereditary spastic paraplegia study that included ALS group for comparison
Zucchi et al. (2018)[89] PMID: 30428468	Prospective	ALS, primary lateral sclerosis, hereditary spastic paraplegia, and healthy controls	Italy	CSF and serum pNfH	ELISA	39	Primarily an upper motor syndrome study

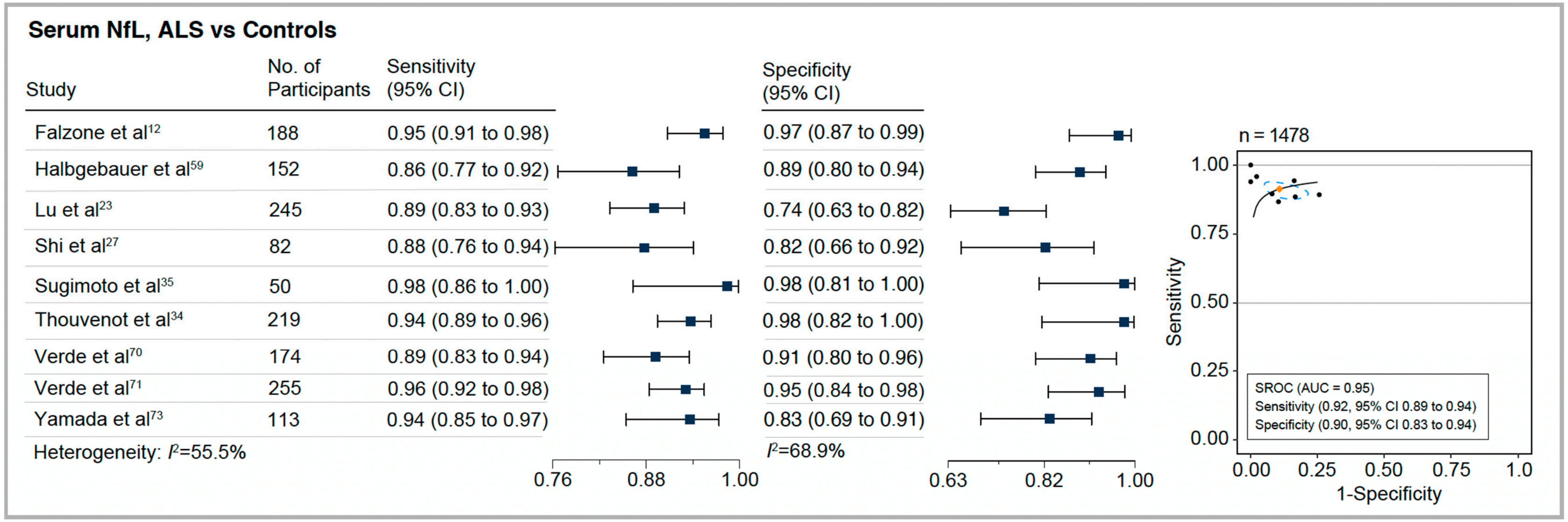

Figure S1. Forest Plots and Receiver Operating Characteristics Curves for the Diagnostic Accuracy of Serum NfL. Forest plots of sensitivity, specificity, and Summary Receiver Operating Characteristic (SROC) and their confidence intervals are presented. Each individual dot represents a unique study. The orange diamond represents the summary estimate of sensitivity and false-positive rate (1-specificity), and the dotted circle represents the 95% confidence region. On top of the SROC, "n" represents the total number of participants in the analyses.

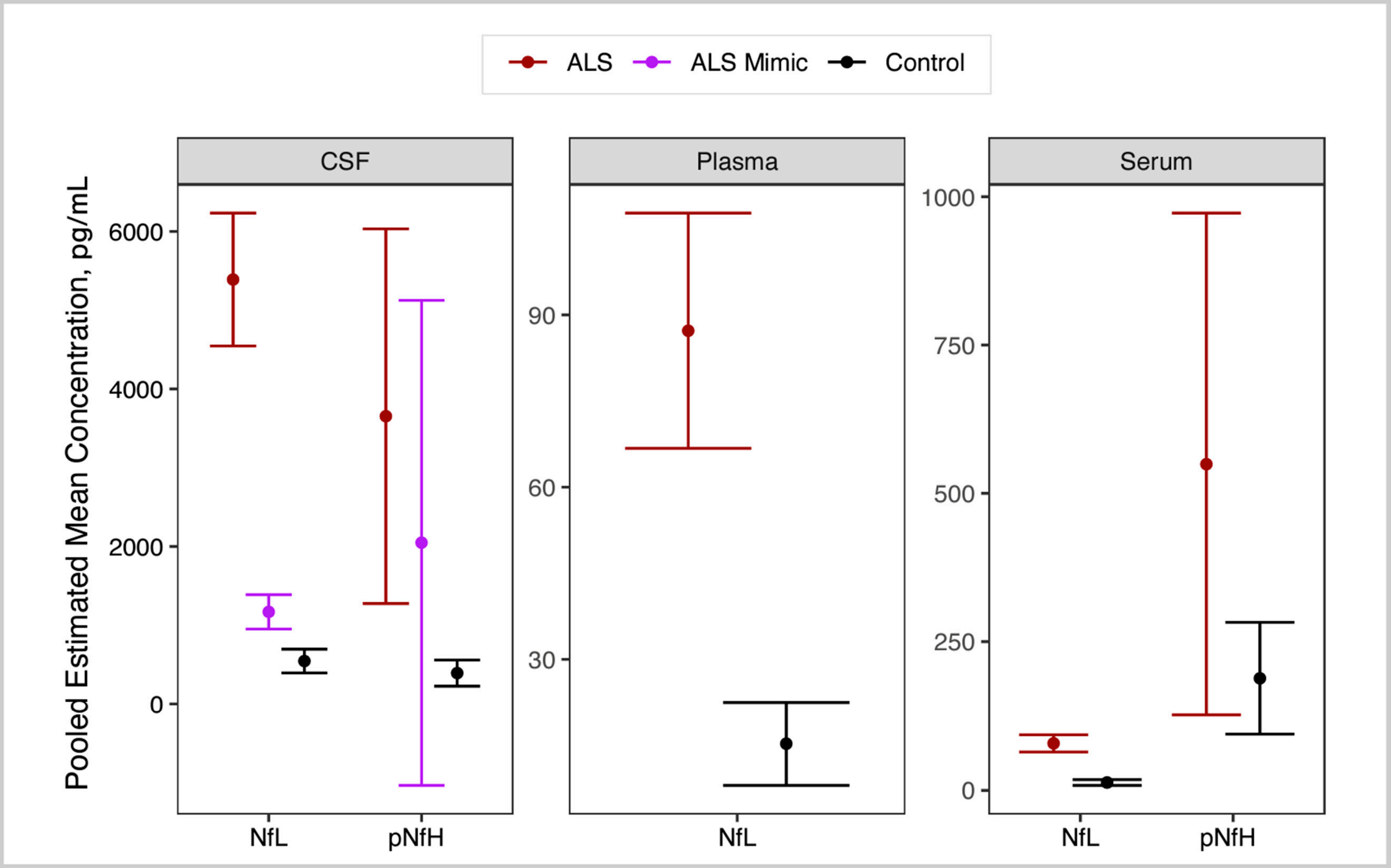

Figure S2. Group Differences in Biomarkers Between ALS, ALS Mimic, and Controls.

The pooled mean concentration and spread (confidence interval) are reported for NfL and pNfH in patients with ALS, ALS mimics, and controls.

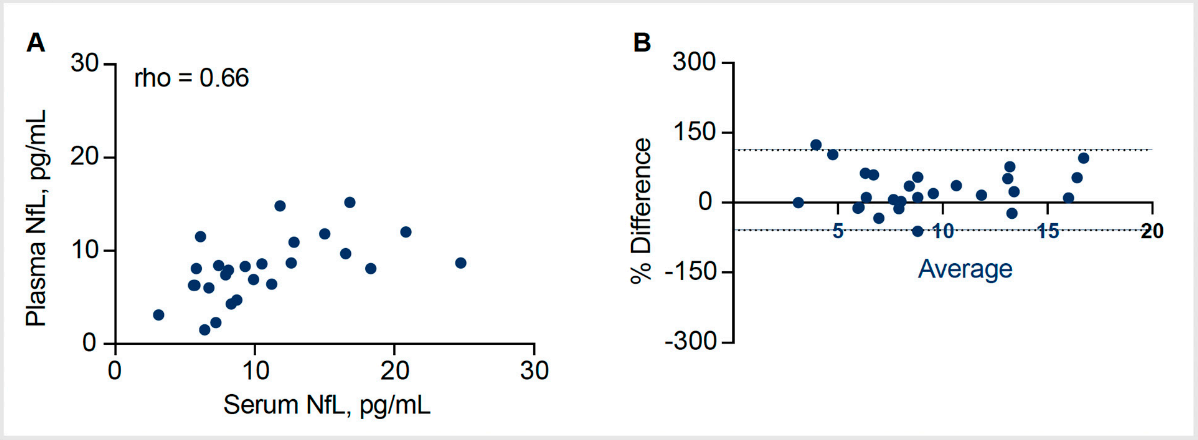

Figure S3. Correlation Plasma and Serum NfL.

(**A**) shows the correlation between plasma and serum NfL. (**B**) shows the Bland-Altman agreement plot of the plasma and serum NfL. Single dots indicate NfL in plasma and serum with their mean concentrations on the x-axis and the difference in concentration between the two sources on the y-axis. The dashed horizontal lines indicate the upper and lower 95% confidence intervals for the mean difference between plasma and serum concentration.

Correlation CSF and Plasma pNfH

Study	*r* (95% CI)
Boylan et al (2013)	0.47 (0.03 to 0.76)
Escal et al (2022)	0.47 (-0.44 to 0.90)
Li et al (2016)	0.71 (0.54 to 0.83)
**Overall**	**0.64 (0.47 to 0.77)**

I^2=0.04%

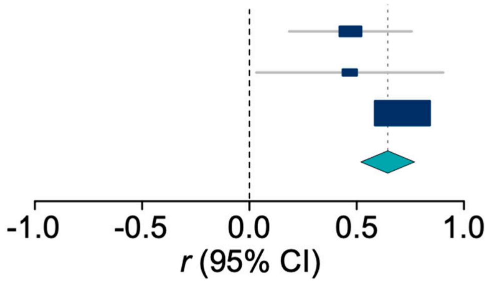

Figure S4. Meta-analysis of Correlation CSF and Plasma pNfH.

The correlations were calculated using the Spearman's rank correlation. Markers indicate estimates, with the size of the marker indicating weight; horizontal lines represent 95% CIs; diamond represent summary estimate, with the outer points indicating 95% confidence intervals.

[c]Mayo cohort (4 month)[5]

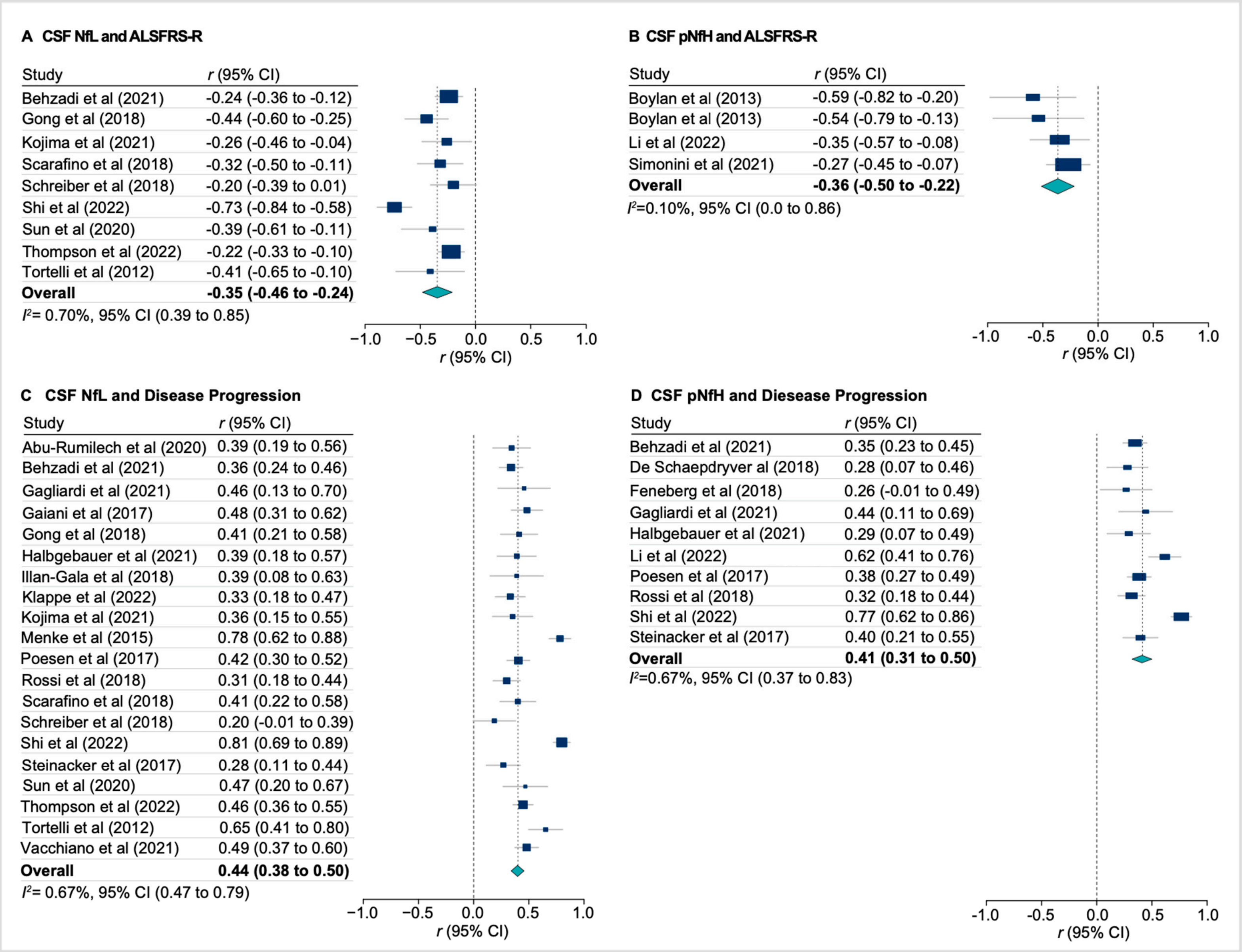
A CSF NfL and ALSFRS-R
Study
r (95% CI)
Behzadi et al (2021) -0.24 (-0.36 to -0.12)
Gong et al (2018) -0.44 (-0.60 to -0.25)
Kojima et al (2021) -0.26 (-0.46 to -0.04)
Scarafino et al (2018) -0.32 (-0.50 to -0.11)
Schreiber et al (2018) -0.20 (-0.39 to 0.01)
Shi et al (2022) -0.73 (-0.84 to -0.58)
Sun et al (2020) -0.39 (-0.61 to -0.11)
Thompson et al (2022) -0.22 (-0.33 to -0.10)
Tortelli et al (2012) -0.41 (-0.65 to -0.10)
Overall -0.35 (-0.46 to -0.24)
I^2= 0.70%, 95% CI (0.39 to 0.85)
−1.0 −0.5 0.0 0.5 1.0
r (95% CI)
B CSF pNfH and ALSFRS-R
Study
r (95% CI)
Boylan et al (2013) -0.59 (-0.82 to -0.20)
Boylan et al (2013) -0.54 (-0.79 to -0.13)
Li et al (2022) -0.35 (-0.57 to -0.08)
Simonini et al (2021) -0.27 (-0.45 to -0.07)
Overall -0.36 (-0.50 to -0.22)
I^2=0.10%, 95% CI (0.0 to 0.86)
-1.0 -0.5 0.0 0.5 1.0
r (95% CI)
C CSF NfL and Disease Progression
Study
r (95% CI)
Abu-Rumilech et al (2020) 0.39 (0.19 to 0.56)
Behzadi et al (2021) 0.36 (0.24 to 0.46)
Gagliardi et al (2021) 0.46 (0.13 to 0.70)
Gaiani et al (2017) 0.48 (0.31 to 0.62)
Gong et al (2018) 0.41 (0.21 to 0.58)
Halbgebauer et al (2021) 0.39 (0.18 to 0.57)
Illan-Gala et al (2018) 0.39 (0.08 to 0.63)
Klappe et al (2022) 0.33 (0.18 to 0.47)
Kojima et al (2021) 0.36 (0.15 to 0.55)
Menke et al (2015) 0.78 (0.62 to 0.88)
Poesen et al (2017) 0.42 (0.30 to 0.52)
Rossi et al (2018) 0.31 (0.18 to 0.44)
Scarafino et al (2018) 0.41 (0.22 to 0.58)
Schreiber et al (2018) 0.20 (-0.01 to 0.39)
Shi et al (2022) 0.81 (0.69 to 0.89)
Steinacker et al (2017) 0.28 (0.11 to 0.44)
Sun et al (2020) 0.47 (0.20 to 0.67)
Thompson et al (2022) 0.46 (0.36 to 0.55)
Tortelli et al (2012) 0.65 (0.41 to 0.80)
Vacchiano et al (2021) 0.49 (0.37 to 0.60)
Overall 0.44 (0.38 to 0.50)
I^2= 0.67%, 95% CI (0.47 to 0.79)
−1.0 −0.5 0.0 0.5 1.0
r (95% CI)
D CSF pNfH and Diesease Progression
Study
r (95% CI)
Behzadi et al (2021) 0.35 (0.23 to 0.45)
De Schaepdryver al (2018) 0.28 (0.07 to 0.46)
Feneberg et al (2018) 0.26 (-0.01 to 0.49)
Gagliardi et al (2021) 0.44 (0.11 to 0.69)
Halbgebauer et al (2021) 0.29 (0.07 to 0.49)
Li et al (2022) 0.62 (0.41 to 0.76)
Poesen et al (2017) 0.38 (0.27 to 0.49)
Rossi et al (2018) 0.32 (0.18 to 0.44)
Shi et al (2022) 0.77 (0.62 to 0.86)
Steinacker et al (2017) 0.40 (0.21 to 0.55)
Overall 0.41 (0.31 to 0.50)
I^2=0.67%, 95% CI (0.37 to 0.83)
-1.0 -0.5 0.0 0.5 1.0
r (95% CI)

Figure S5. Meta-analysis of Relationships of pNfH and NfL Measured in CSF and ALSFRS-R and Disease Progression. (**A**) Correlation cerebrospinal fluid (CSF) NfL with ALS Functional Rating Scale (ALSFRS-R). (**B**) Correlation CSF pNfH with ALSFRS-R. (**D**) Correlation CSF NfL with disease progression. (**D**) Correlation CSF pNfH with disease progression. All correlations shown are Spearman's rank correlations. Markers indicate estimates, with the size of the marker indicating weight; horizontal lines represent 95% confidence intervals; diamonds represent summary estimates, with the outer points indicating 95% confidence intervals.

Survival Analysis blood NfL

Study	No. of Participants	*r* (95% CI)
Behazadi et al (2021)	213	-0.42 (-0.31 to 0.52)
Benatar et al (2020)	229	-0.21 (-0.08 to -0.21)
Verde et al (2023)	124	-0.35 (-0.19 to -0.50)
Witzel et al (2021)	128	-0.53 (-0.39 to -0.50)
**Overall**	**694**	**-0.38 (-0.24 to -0.51)**

I^2=0.77%, 95% CI (0.37 to 0.92)

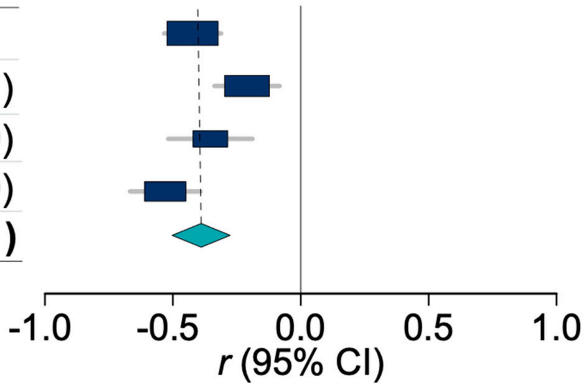

Figure S6. Meta-analysis of Correlation Between Serum NfL and Survival Time.

All correlations are Spearman's rank correlations. Markers indicate estimates, with the size of the marker indicating weight; horizontal lines represent 95% confidence intervals; diamonds represent summary estimates, with the outer points indicating 95% confidence intervals.

Figure S7. Risk of Bias and Concerns for Applicability Assessment

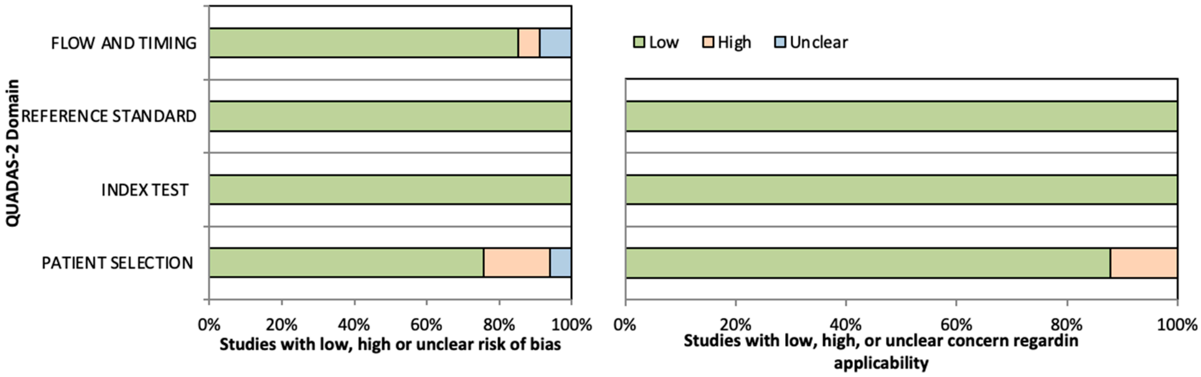

Table S3. Sample Size Per Group for Various Effect Estimates			
Treatment Effectiveness (%)	Sample Size Per Group	Outcome	Cohen's *d*
10	137	CSF NfL	3.4
15	61	CSF NfL	3.4
20	34	CSF NfL	3.4
25	22	CSF NfL	3.4
30	15	CSF NfL	3.4
35	11	CSF NfL	3.4
40	9	CSF NfL	3.4
45	7	CSF NfL	3.4
50	5	CSF NfL	3.4
10	1425	CSF PNFH	1.0
15	633	CSF PNFH	1.0
20	356	CSF PNFH	1.0
25	228	CSF PNFH	1.0
30	158	CSF PNFH	1.0
35	116	CSF PNFH	1.0
40	89	CSF PNFH	1.0
45	70	CSF PNFH	1.0
50	57	CSF PNFH	1.0
10	99	Serum NfL	4.0
15	44	Serum NfL	4.0
20	25	Serum NfL	4.0
25	16	Serum NfL	4.0
30	11	Serum NfL	4.0
35	8	Serum NfL	4.0
40	6	Serum NfL	4.0
45	5	Serum NfL	4.0
50	4	Serum NfL	4.0
10	2020	Serum PNFH	0.9
15	898	Serum PNFH	0.9
20	505	Serum PNFH	0.9
25	323	Serum PNFH	0.9
30	224	Serum PNFH	0.9
35	165	Serum PNFH	0.9
40	126	Serum PNFH	0.9
45	100	Serum PNFH	0.9
50	81	Serum PNFH	0.9
10	216	Plasma NfL	2.7

15	96	Plasma NfL	2.7
20	54	Plasma NfL	2.7
25	35	Plasma NfL	2.7
30	24	Plasma NfL	2.7
35	18	Plasma NfL	2.7
40	14	Plasma NfL	2.7
45	11	Plasma NfL	2.7
50	9	Plasma NfL	2.7

Table S4. Sample Size for Paired Assessments at Various Effect Sizes using Longitudinal Studies[30, 70]

Cohen's *d*	Sample Size Per Group	Outcome
0.1	825	Serum NfL
0.2 (small effect)	208	Serum NfL
0.3	95	Serum NfL
0.4	55	Serum NfL
0.5 (moderate effect)	36	Serum NfL
0.6	26	Serum NfL
0.7	20	Serum NfL
0.8 (large effect)	16	Serum NfL
0.9	13	Serum NfL

Table S5. Review of Studies of Neurofilaments in FTD-ALS Spectrum							
**Study**	**Genotypes**	**Study Design**	***N*[a]**	**Biomarker**	**ALS compared to FTD[b]**	**ALS compared to ALS-FTD**	**Biomarker Differences Across Genotype**
Saracino et al (2021)[65] PMID: 34349004	*C9orf72, GRN*	Prospective	102	Plasma NfL	Yes	-	Higher plasma NfL in *GRN* than *C9ORF72* mutation carriers
Escal et al (2022)[10] PMID: 34313819	*C9orf72, GRN*	Retrospective	81	Plasma NfL	Yes	No	Higher plasma NfL in *GRN* than *C9ORF72* mutation carriers.
Escal et al (2022)[10] PMID: 34313819	*C9orf72, GRN*	Retrospective	81	Plasma pNfH	Yes	Yes	No
Gaiani et al (2017)[14] PMID: 28264096	*C9orf72*	Retrospective	114	CSF NfL	Yes	-	-
Halbgebauer et al (2022)[59] PMID: 34417339	-	Prospective	88	CSF NfL	No	-	-
Halbgebauer et al (2022)[59] PMID: 34417339	-	Prospective	88	CSF pNfH	Yes	-	-
Halbgebauer et al (2022)[59] PMID: 34417339	-	Prospective	88	Serum NfL	No	-	-

Halbgebauer et al (2022)[59] PMID: 34417339	-	Prospective	88	Serum pNfH	Yes	-	-

Illan-Gala et al (2018)[60] PMID: 30291183	*C9orf72, GRN, VCP, TARDBP*	Prospective	124	CSF NfL	Yes	-	-
Verde et al (2019)[70] PMID: 30309882	*C9orf72, SOD1*	Prospective	136	Serum NfL	Yes	-	No difference in serum NfL between *C9ORF72* and *SOD1* mutation carriers

[a]The *N* is calculated only for the groups compared.
[b]Behavioral variant

Table S6. Review of Studies of Neurofilaments in ALS patients with genetic forms of disease.

**Study**	**Genotypes**	**Study Design**	***N*[a]**	**Biomarker**	**Compared to Controls**	**Biomarker Differences Across Genotype**
Benatar et al (2018)[7] PMID: 30014505	*SOD1, FUS, C9orf72*	Prospective	94	Serum NfL	No difference	Not assessed
Benatar et al (2018)[7] PMID: 30014505	*SOD1, FUS, C9orf72*	Prospective	63	CSF NfL	No difference	Not assessed
Poesen et al (2017)[25] PMID: 28500227	*C9orf72*	Prospective	8	CSF pNfH	No difference	Not assessed
Weydt et al (2016)[36] PMID: 26528863	*SOD1, FUS, C9orf72, TARDBP*	Prospective	7	CSF NfL	No difference	Not assessed
Weydt et al (2016)[36] PMID: 26528863	*SOD1, FUS, C9orf72, TARDBP*	Prospective	9	CSF pNfH	No difference	Not assessed
Weydt et al (2016)[36] PMID: 26528863	*SOD1, FUS, C9orf72, TARDBP*	Prospective	11	Blood NfL	No difference	Not assessed
Gendron et al (2017)[57] PMID: 28628244	*C9orf72*	Prospective	135	CSF pNfH	Yes	Increased CSF pNfH in *C9*-ALS carriers vs non-carriers
Saracino et al (2021)[65] PMID: 34349004	*C9orf72, GRN*	Prospective	102	Plasma NfL	Yes	Higher plasma NfL in *GRN* than *C9orf72*
Verde et al (2019)[70] PMID: 30309882	*C9orf72, SOD1*	Prospective	136	Serum NfL	Yes	No difference in serum NfL between

						C9orf72 and *SOD1* mutation carriers
Zetterberg et al (2007)[74] PMID: 17903209	*SOD1*	Retrospective	79	CSF NfL	Yes	Lower CSF NfL in *SOD1* mutation carriers than *SOD1* wt
[a]The *N* is calculated only for the groups compared.						

Table S7. Review of Studies of Neurofilaments in Presymptomatic ALS patients						
**Study**	**Genotypes**	**Study Design**	***N*[a]**	**Biomarker**	**Presymptomatic Compared to Controls**	**Pre-Symptomatic Longitudinal Increase**
Benatar et al (2018)[7] PMID: 30014505	*SOD1, FUS, C9orf72*	Prospective	94	CSF and serum NfL	No difference	Yes (except for pNfH)
Benatar et al (2019)[3] PMID: 31432691	*SOD1, FUS, C9orf72*	Prospective	115	CSF NfL, serum NfL and CSF pNfH and serum pNfH	No difference	Yes (except for pNfH)
Poesen et al (2017)[25] PMID: 28500227	*C9orf72*	Prospective	8	CSF NfL and CSF pNfH	No difference	Not assessed
Weydt et al (2016)[36] PMID: 26528863	*SOD1, FUS, C9orf72, TARDBP*	Prospective	12	CSF and blood NfL and CSF pNfH	No difference	Not assessed
Gendron et al (2017) PMID: 28628244	*C9orf72*	Prospective	135	CSF pNfH	Yes	Increased CSF pNfH in *C9*-ALS carriers vs non-carriers
Saracino et al (2021) PMID: 34349004	*C9orf72, GRN*	Prospective	28	Plasma NfL	No	Yes (in 4 individuals who had longitudinal samples)
[a]The *N* is calculated only for the groups compared.						

REFERENCES

1. Page MJ, McKenzie JE, Bossuyt PM, et al. The PRISMA 2020 statement: an updated guideline for reporting systematic reviews. Rev Esp Cardiol (Engl Ed) 2021;74:790-799.
2. McInnes MDF, Moher D, Thombs BD, et al. Preferred Reporting Items for a Systematic Review and Meta-analysis of Diagnostic Test Accuracy Studies: The PRISMA-DTA Statement. JAMA 2018;319:388-396.
3. Benatar M, Wuu J, Lombardi V, et al. Neurofilaments in pre-symptomatic ALS and the impact of genotype. Amyotroph Lateral Scler Frontotemporal Degener 2019;20:538-548.
4. Benatar M, Zhang L, Wang L, et al. Validation of serum neurofilaments as prognostic and potential pharmacodynamic biomarkers for ALS. Neurology 2020;95:e59-e69.
5. Boylan KB, Glass JD, Crook JE, et al. Phosphorylated neurofilament heavy subunit (pNF-H) in peripheral blood and CSF as a potential prognostic biomarker in amyotrophic lateral sclerosis. J Neurol Neurosurg Psychiatry 2013;84:467-472.
6. Brettschneider J, Petzold A, Sussmuth SD, Ludolph AC, Tumani H. Axonal damage markers in cerebrospinal fluid are increased in ALS. Neurology 2006;66:852-856.
7. Benatar M, Wuu J, Andersen PM, Lombardi V, Malaspina A. Neurofilament light: A candidate biomarker of presymptomatic amyotrophic lateral sclerosis and phenoconversion. Ann Neurol 2018;84:130-139.
8. Bjornevik K, O'Reilly EJ, Molsberry S, et al. Prediagnostic Neurofilament Light Chain Levels in Amyotrophic Lateral Sclerosis. Neurology 2021;97:e1466-1474.
9. Dreger M, Steinbach R, Gaur N, et al. Cerebrospinal Fluid Neurofilament Light Chain (NfL) Predicts Disease Aggressiveness in Amyotrophic Lateral Sclerosis: An Application of the D50 Disease Progression Model. Front Neurosci 2021;15:651651.
10. Escal J, Fourier A, Formaglio M, et al. Comparative diagnosis interest of NfL and pNfH in CSF and plasma in a context of FTD-ALS spectrum. J Neurol 2022;269:1522-1529.
11. Falzone YM, Domi T, Agosta F, et al. Serum phosphorylated neurofilament heavy-chain levels reflect phenotypic heterogeneity and are an independent predictor of survival in motor neuron disease. J Neurol 2020;267:2272-2280.
12. Falzone YM, Domi T, Mandelli A, et al. Integrated evaluation of a panel of neurochemical biomarkers to optimize diagnosis and prognosis in amyotrophic lateral sclerosis. Eur J Neurol 2022;29:1930-1939.
13. Gagliardi D, Faravelli I, Meneri M, et al. Diagnostic and prognostic value of CSF neurofilaments in a cohort of patients with motor neuron disease: A cross-sectional study. J Cell Mol Med 2021;25:3765-3771.
14. Gaiani A, Martinelli I, Bello L, et al. Diagnostic and Prognostic Biomarkers in Amyotrophic Lateral Sclerosis: Neurofilament Light Chain Levels in Definite Subtypes of Disease. JAMA Neurol 2017;74:525-532.
15. Ganesalingam J, An J, Bowser R, Andersen PM, Shaw CE. pNfH is a promising biomarker for ALS. Amyotroph Lateral Scler Frontotemporal Degener 2013;14:146-149.
16. Ganesalingam J, An J, Shaw CE, Shaw G, Lacomis D, Bowser R. Combination of neurofilament heavy chain and complement C3 as CSF biomarkers for ALS. J Neurochem 2011;117:528-537.
17. Gong ZY, Lv GP, Gao LN, Lu Y, Guo J, Zang DW. Neurofilament Subunit L Levels in the Cerebrospinal Fluid and Serum of Patients with Amyotrophic Lateral Sclerosis. Neurodegener Dis 2018;18:165-172.

18. Goncalves M, Tillack L, de Carvalho M, Pinto S, Conradt HS, Costa J. Phosphoneurofilament heavy chain and N-glycomics from the cerebrospinal fluid in amyotrophic lateral sclerosis. Clin Chim Acta 2015;438:342-349.
19. Klappe U, Chamoun S, Shen Q, et al. Cardiac troponin T is elevated and increases longitudinally in ALS patients. Amyotroph Lateral Scler Frontotemporal Degener 2022;23:58-65.
20. Kojima Y, Kasai T, Noto YI, et al. Amyotrophic lateral sclerosis: Correlations between fluid biomarkers of NfL, TDP-43, and tau, and clinical characteristics. PLoS One 2021;16:e0260323.
21. Li DW, Ren H, Jeromin A, et al. Diagnostic Performance of Neurofilaments in Chinese Patients With Amyotrophic Lateral Sclerosis: A Prospective Study. Front Neurol 2018;9:726.
22. Li S, Ren Y, Zhu W, Yang F, Zhang X, Huang X. Phosphorylated neurofilament heavy chain levels in paired plasma and CSF of amyotrophic lateral sclerosis. J Neurol Sci 2016;367:269-274.
23. Lu CH, Macdonald-Wallis C, Gray E, et al. Neurofilament light chain: A prognostic biomarker in amyotrophic lateral sclerosis. Neurology 2015;84:2247-2257.
24. Lu CH, Petzold A, Topping J, et al. Plasma neurofilament heavy chain levels and disease progression in amyotrophic lateral sclerosis: insights from a longitudinal study. J Neurol Neurosurg Psychiatry 2015;86:565-573.
25. Poesen K, De Schaepdryver M, Stubendorff B, et al. Neurofilament markers for ALS correlate with extent of upper and lower motor neuron disease. Neurology 2017;88:2302-2309.
26. Reijn TS, Abdo WF, Schelhaas HJ, Verbeek MM. CSF neurofilament protein analysis in the differential diagnosis of ALS. J Neurol 2009;256:615-619.
27. Shi J, Qin X, Chang X, Wang H, Guo J, Zhang W. Neurofilament markers in serum and cerebrospinal fluid of patients with amyotrophic lateral sclerosis. J Cell Mol Med 2022;26:583-587.
28. Simonini C, Zucchi E, Bedin R, et al. CSF Heavy Neurofilament May Discriminate and Predict Motor Neuron Diseases with Upper Motor Neuron Involvement. Biomedicines 2021;9.
29. Steinacker P, Feneberg E, Weishaupt J, et al. Neurofilaments in the diagnosis of motoneuron diseases: a prospective study on 455 patients. J Neurol Neurosurg Psychiatry 2016;87:12-20.
30. Steinacker P, Huss A, Mayer B, et al. Diagnostic and prognostic significance of neurofilament light chain NF-L, but not progranulin and S100B, in the course of amyotrophic lateral sclerosis: Data from the German MND-net. Amyotroph Lateral Scler Frontotemporal Degener 2017;18:112-119.
31. Sun Q, Zhao X, Li S, et al. CSF Neurofilament Light Chain Elevation Predicts ALS Severity and Progression. Front Neurol 2020;11:919.
32. Thompson AG, Gray E, Bampton A, Raciborska D, Talbot K, Turner MR. CSF chitinase proteins in amyotrophic lateral sclerosis. J Neurol Neurosurg Psychiatry 2019;90:1215-1220.
33. Thompson AG, Gray E, Verber N, et al. Multicentre appraisal of amyotrophic lateral sclerosis biofluid biomarkers shows primacy of blood neurofilament light chain. Brain Commun 2022;4:fcac029.
34. Thouvenot E, Demattei C, Lehmann S, et al. Serum neurofilament light chain at time of diagnosis is an independent prognostic factor of survival in amyotrophic lateral sclerosis. Eur J Neurol 2020;27:251-257.

35. Sugimoto K, Han Y, Song Y, Gao Y. Correlational Analysis of ALS Progression and Serum NfL Measured by Simoa Assay in Chinese Patients. Front Neurol 2020;11:579094.
36. Weydt P, Oeckl P, Huss A, et al. Neurofilament levels as biomarkers in asymptomatic and symptomatic familial amyotrophic lateral sclerosis. Ann Neurol 2016;79:152-158.
37. Zecca C, Dell'Abate MT, Pasculli G, et al. Role of plasma phosphorylated neurofilament heavy chain (pNfH) in amyotrophic lateral sclerosis. J Cell Mol Med 2022;26:3608-3615.
38. Cedarbaum JM, Stambler N, Malta E, et al. The ALSFRS-R: a revised ALS functional rating scale that incorporates assessments of respiratory function. BDNF ALS Study Group (Phase III). J Neurol Sci 1999;169:13-21.
39. Kimura F, Fujimura C, Ishida S, et al. Progression rate of ALSFRS-R at time of diagnosis predicts survival time in ALS. Neurology 2006;66:265-267.
40. Gebru T, Lentiro K. The impact of community-based health insurance on health-related quality of life and associated factors in Ethiopia: a comparative cross-sectional study. Health Qual Life Outcomes 2018;16:110.
41. Whiting PF, Rutjes AW, Westwood ME, et al. QUADAS-2: a revised tool for the quality assessment of diagnostic accuracy studies. Ann Intern Med 2011;155:529-536.
42. Rissin DM, Kan CW, Campbell TG, et al. Single-molecule enzyme-linked immunosorbent assay detects serum proteins at subfemtomolar concentrations. Nat Biotechnol 2010;28:595-599.
43. Shahim P, Gren M, Liman V, et al. Serum neurofilament light protein predicts clinical outcome in traumatic brain injury. Sci Rep 2016;6:36791.
44. McGrath S, Sohn H, Steele R, Benedetti A. Meta-analysis of the difference of medians. Biom J 2020;62:69-98.
45. Wan X, Wang W, Liu J, Tong T. Estimating the sample mean and standard deviation from the sample size, median, range and/or interquartile range. BMC Med Res Methodol 2014;14:135.
46. Cash DM, Rohrer JD, Ryan NS, Ourselin S, Fox NC. Imaging endpoints for clinical trials in Alzheimer's disease. Alzheimers Res Ther 2014;6:87.
47. Hua X, Lee S, Yanovsky I, et al. Optimizing power to track brain degeneration in Alzheimer's disease and mild cognitive impairment with tensor-based morphometry: an ADNI study of 515 subjects. Neuroimage 2009;48:668-681.
48. Abu-Rumeileh S, Vacchiano V, Zenesini C, et al. Diagnostic-prognostic value and electrophysiological correlates of CSF biomarkers of neurodegeneration and neuroinflammation in amyotrophic lateral sclerosis. J Neurol 2020;267:1699-1708.
49. Behzadi A, Pujol-Calderon F, Tjust AE, et al. Neurofilaments can differentiate ALS subgroups and ALS from common diagnostic mimics. Sci Rep 2021;11:22128.
50. Costa J, Gromicho M, Pronto-Laborinho A, et al. Cerebrospinal Fluid Chitinases as Biomarkers for Amyotrophic Lateral Sclerosis. Diagnostics (Basel) 2021;11.
51. Chen X, Chen Y, Wei Q, et al. Assessment of a multiple biomarker panel for diagnosis of amyotrophic lateral sclerosis. BMC Neurol 2016;16:173.
52. De Schaepdryver M, Jeromin A, Gille B, et al. Comparison of elevated phosphorylated neurofilament heavy chains in serum and cerebrospinal fluid of patients with amyotrophic lateral sclerosis. J Neurol Neurosurg Psychiatry 2018;89:367-373.
53. De Schaepdryver M, Goossens J, De Meyer S, et al. Serum neurofilament heavy chains as early marker of motor neuron degeneration. Ann Clin Transl Neurol 2019;6:1971-1979.

54. De Schaepdryver M, Lunetta C, Tarlarini C, et al. Neurofilament light chain and C reactive protein explored as predictors of survival in amyotrophic lateral sclerosis. J Neurol Neurosurg Psychiatry 2020;91:436-437.
55. De Schaepdryver M, Masrori P, Van Damme P, Poesen K. Effect of neurofilament analysis on the diagnostic delay in amyotrophic lateral sclerosis. CNS Neurosci Ther 2023;29:70-77.
56. Feneberg E, Oeckl P, Steinacker P, et al. Multicenter evaluation of neurofilaments in early symptom onset amyotrophic lateral sclerosis. Neurology 2018;90:e22-e30.
57. Gendron TF, Group CONS, Daughrity LM, et al. Phosphorylated neurofilament heavy chain: A biomarker of survival for C9ORF72-associated amyotrophic lateral sclerosis. Ann Neurol 2017;82:139-146.
58. Gille B, De Schaepdryver M, Goossens J, et al. Serum neurofilament light chain levels as a marker of upper motor neuron degeneration in patients with Amyotrophic Lateral Sclerosis. Neuropathol Appl Neurobiol 2019;45:291-304.
59. Halbgebauer S, Steinacker P, Verde F, et al. Comparison of CSF and serum neurofilament light and heavy chain as differential diagnostic biomarkers for ALS. J Neurol Neurosurg Psychiatry 2022;93:68-74.
60. Illan-Gala I, Alcolea D, Montal V, et al. CSF sAPPbeta, YKL-40, and NfL along the ALS-FTD spectrum. Neurology 2018;91:e1619-e1628.
61. Kasai T, Kojima Y, Ohmichi T, et al. Combined use of CSF NfL and CSF TDP-43 improves diagnostic performance in ALS. Ann Clin Transl Neurol 2019;6:2489-2502.
62. Menke RA, Gray E, Lu CH, et al. CSF neurofilament light chain reflects corticospinal tract degeneration in ALS. Ann Clin Transl Neurol 2015;2:748-755.
63. Rosengren LE, Karlsson JE, Karlsson JO, Persson LI, Wikkelso C. Patients with amyotrophic lateral sclerosis and other neurodegenerative diseases have increased levels of neurofilament protein in CSF. J Neurochem 1996;67:2013-2018.
64. Rossi D, Volanti P, Brambilla L, Colletti T, Spataro R, La Bella V. CSF neurofilament proteins as diagnostic and prognostic biomarkers for amyotrophic lateral sclerosis. J Neurol 2018;265:510-521.
65. Saracino D, Dorgham K, Camuzat A, et al. Plasma NfL levels and longitudinal change rates in C9orf72 and GRN-associated diseases: from tailored references to clinical applications. J Neurol Neurosurg Psychiatry 2021;92:1278-1288.
66. Scarafino A, D'Errico E, Introna A, et al. Diagnostic and prognostic power of CSF Tau in amyotrophic lateral sclerosis. J Neurol 2018;265:2353-2362.
67. Schreiber S, Spotorno N, Schreiber F, et al. Significance of CSF NfL and tau in ALS. J Neurol 2018;265:2633-2645.
68. Tortelli R, Ruggieri M, Cortese R, et al. Elevated cerebrospinal fluid neurofilament light levels in patients with amyotrophic lateral sclerosis: a possible marker of disease severity and progression. Eur J Neurol 2012;19:1561-1567.
69. Vacchiano V, Mastrangelo A, Zenesini C, et al. Plasma and CSF Neurofilament Light Chain in Amyotrophic Lateral Sclerosis: A Cross-Sectional and Longitudinal Study. Front Aging Neurosci 2021;13:753242.
70. Verde F, Steinacker P, Weishaupt JH, et al. Neurofilament light chain in serum for the diagnosis of amyotrophic lateral sclerosis. J Neurol Neurosurg Psychiatry 2019;90:157-164.
71. Verde F, Milone I, Colombo E, et al. Phenotypic correlates of serum neurofilament light chain levels in amyotrophic lateral sclerosis. Front Aging Neurosci 2023;15:1132808.

72. Witzel S, Frauhammer F, Steinacker P, et al. Neurofilament light and heterogeneity of disease progression in amyotrophic lateral sclerosis: development and validation of a prediction model to improve interventional trials. Transl Neurodegener 2021;10:31.
73. Yamada S, Hashizume A, Hijikata Y, et al. Ratio of urinary N-terminal titin fragment to urinary creatinine is a novel biomarker for amyotrophic lateral sclerosis. J Neurol Neurosurg Psychiatry 2021;92:1072-1079.
74. Zetterberg H, Jacobsson J, Rosengren L, Blennow K, Andersen PM. Cerebrospinal fluid neurofilament light levels in amyotrophic lateral sclerosis: impact of SOD1 genotype. Eur J Neurol 2007;14:1329-1333.
75. Zhang L, Ji T, Wu C, et al. Serum Neurofilament Light Chain Levels May Be a Marker of Lower Motor Neuron Damage in Amyotrophic Lateral Sclerosis. Front Neurol 2022;13:833507.
76. Brodovitch A, Boucraut J, Delmont E, et al. Combination of serum and CSF neurofilament-light and neuroinflammatory biomarkers to evaluate ALS. Sci Rep 2021;11:703.
77. Delaby C, Alcolea D, Carmona-Iragui M, et al. Differential levels of Neurofilament Light protein in cerebrospinal fluid in patients with a wide range of neurodegenerative disorders. Sci Rep 2020;10:9161.
78. Gray E, Oeckl P, Amador MDM, et al. A multi-center study of neurofilament assay reliability and inter-laboratory variability. Amyotroph Lateral Scler Frontotemporal Degener 2020;21:452-458.
79. Huang F, Zhu Y, Hsiao-Nakamoto J, et al. Longitudinal biomarkers in amyotrophic lateral sclerosis. Ann Clin Transl Neurol 2020;7:1103-1116.
80. Ingannato A, Bagnoli S, Mazzeo S, et al. Neurofilament Light Chain and Intermediate HTT Alleles as Combined Biomarkers in Italian ALS Patients. Front Neurosci 2021;15:695049.
81. Kaiserova M, Grambalova Z, Otruba P, et al. Cerebrospinal fluid levels of chromogranin A and phosphorylated neurofilament heavy chain are elevated in amyotrophic lateral sclerosis. Acta Neurol Scand 2017;136:360-364.
82. Lombardi V, Querin G, Ziff OJ, et al. Muscle and not neuronal biomarkers correlate with severity in spinal and bulbar muscular atrophy. Neurology 2019;92:e1205-e1211.
83. McCombe PA, Pfluger C, Singh P, Lim CY, Airey C, Henderson RD. Serial measurements of phosphorylated neurofilament-heavy in the serum of subjects with amyotrophic lateral sclerosis. J Neurol Sci 2015;353:122-129.
84. Oeckl P, Jardel C, Salachas F, et al. Multicenter validation of CSF neurofilaments as diagnostic biomarkers for ALS. Amyotroph Lateral Scler Frontotemporal Degener 2016;17:404-413.
85. Pawlitzki M, Schreiber S, Bittner D, et al. CSF Neurofilament Light Chain Levels in Primary Progressive MS: Signs of Axonal Neurodegeneration. Front Neurol 2018;9:1037.
86. Sabbatini D, Raggi F, Ruggero S, et al. Evaluation of peripherin in biofluids of patients with motor neuron diseases. Ann Clin Transl Neurol 2021;8:1750-1754.
87. Tortelli R, Copetti M, Ruggieri M, et al. Cerebrospinal fluid neurofilament light chain levels: marker of progression to generalized amyotrophic lateral sclerosis. Eur J Neurol 2015;22:215-218.
88. Wilke C, Rattay TW, Hengel H, et al. Serum neurofilament light chain is increased in hereditary spastic paraplegias. Ann Clin Transl Neurol 2018;5:876-882.

89. Zucchi E, Bedin R, Fasano A, et al. Cerebrospinal Fluid Neurofilaments May Discriminate Upper Motor Neuron Syndromes: A Pilot Study. Neurodegener Dis 2018;18:255-261.
